# Zeylleucapenoids A–D, Highly Oxygenated Diterpenoids with Anti-Inflammatory Activity from *Leucas zeylanica* (L.) R. Br.

**DOI:** 10.3390/molecules28114472

**Published:** 2023-05-31

**Authors:** Ting Zhao, Xuan Zhang, Xu-Hua Nong, Xue-Ming Zhou, Ru-Ru Chai, Xiao-Bao Li, Guang-Ying Chen

**Affiliations:** 1 Key Laboratory of Tropical Medicinal Resource Chemistry of Ministry of Education Hainan Normal University, Haikou 571158, China; zhaoting19930812@126.com (T.Z.); zxuan0328@163.com (X.Z.); nongxuhua4883@163.com (X.-H.N.); xueming2009211@126.com (X.-M.Z.); 13119000891@163.com (R.-R.C.); 2 Key Laboratory of Tropical Medicinal Plant Chemistry of Hainan Province, College of Chemistry and Chemical Engineering, Hainan Normal University, Haikou 571158, China

**Keywords:** *Leucas zeylanica*, highly oxygenated, diterpenoids, anti-inflammatory activity, molecular docking, zebrafish model

## Abstract

Four previously undescribed highly oxygenated diterpenoids (**1**–**4**), zeylleucapenoids A–D, characterized by halimane and labdane skeletons, were isolated from the aerial parts of *Leucas zeylanica*. Their structures were elucidated primarily via NMR experiments. The absolute configuration of **1** was established using theoretical ECD calculations and X-ray crystallographic analysis, whereas those for **2**–**4** were assigned using theoretical ORD calculations. Zeylleucapenoids A–D were tested for anti-inflammatory activity against nitric oxide (NO) production in RAW264.7 macrophages, of which only **4** showed significant efficacy with an IC_50_ value of 38.45 μM. Further, active compound **4** was also evaluated for the inhibition of the release of pro-inflammatory cytokines TNF-*α* and IL-6 and was found to have a dose-dependent inhibitory effect, while it showed nontoxic activity for zebrafish embryos. A subsequent Western blotting experiment revealed that **4** inhibited the expression of inducible nitric oxide synthase (iNOS) and cyclooxygenase-2 (COX-2). Furthermore, molecular docking analysis indicated that the possible mechanism of action for **4** may be bind to targets via hydrogen and hydrophobic bond interactions.

## 1. Introduction

Inflammation has been closely related to the immune defense response of patients with chronic diseases [1,2]. The discovery of new anti-inflammatory agents gave hope for the treatment of inflammation-linked diseases, such as metabolic syndromes, autoimmune diseases, and so on. Medicinal plants were an important source for the development of lead drugs. Hitherto, investigations of the chemical constituents of medicinal plants have attracted much attention from chemists. Diterpenoids are a large group of naturally occurring chemical constituents found in terrestrial plants, microbes, insects, and marine organisms [3,4], which exhibit a wide variety of bioactivities, such as anti-inflammatory, antimicrobial, antitumor, and analgesic activities [5,6].

*Leucas* species are perennial herbs and distributed mainly in East Africa and the subtropical area of Asia [7]. There are about 125 *Leucas* species globally, of which 7 species grow in southern China. Studies on a few species led to the isolation of diterpenoids as active constituents, which exhibited anti-inflammatory and anti-mycobacterial activities [8,9]. The aerial parts of *L. zeylanica* have been used as folk medicine in treating inflammatory diseases, e.g., pertussis, asthma, headache and indigestion [10].

**Figure 1:**
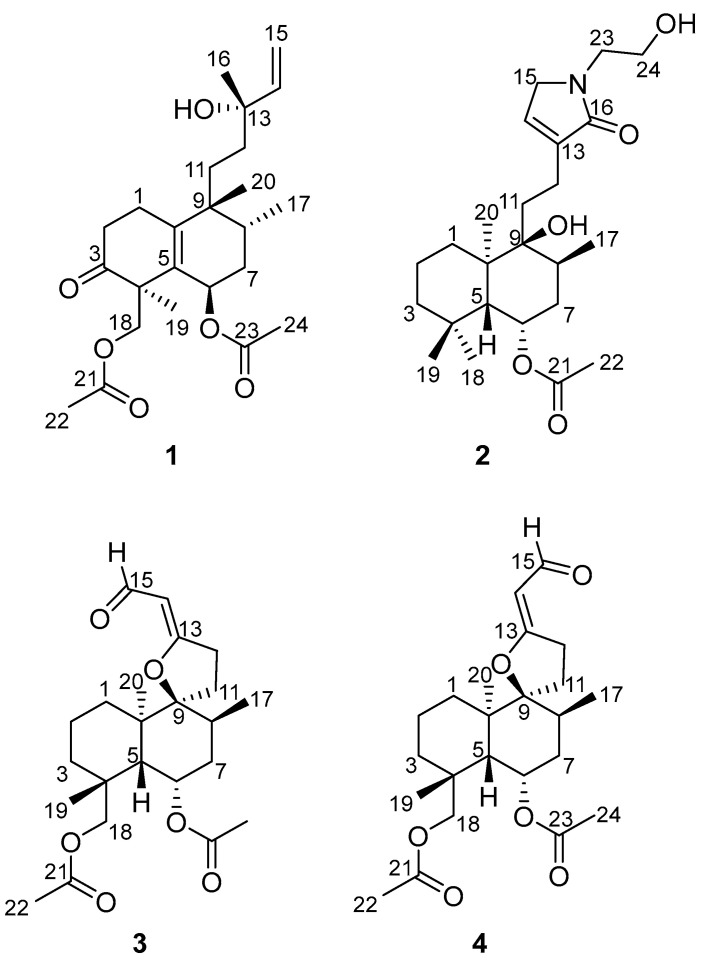
Structures of compounds **1**–**4** from *L. zeylanica*.

Our previous chemical investigations of *L. zeylanica* led to the identification of labdane diterpenoids and flavonoids [11]. In search of novel, bioactive, and structurally diverse natural products from traditional Chinese folk medicine, four undescribed highly oxygenated diterpenoids, compounds **1**–**4**, were isolated from the aerial parts of *L. zeylanica*. Among them, compound **1** was elucidated to be a halimane-type diterpenoid, while **2**–**4** were labdane-type diterpenoids (Figure 1). Here, we report the structures, anti-inflammatory effects and the potential mechanisms of the isolated diterpenoids.

## 2. Results and Discussion

### 2.1. Elucidation of the Chemical Structures of Zeylleucapenoids A–D (***1**–**4***)

**Figure 2:**
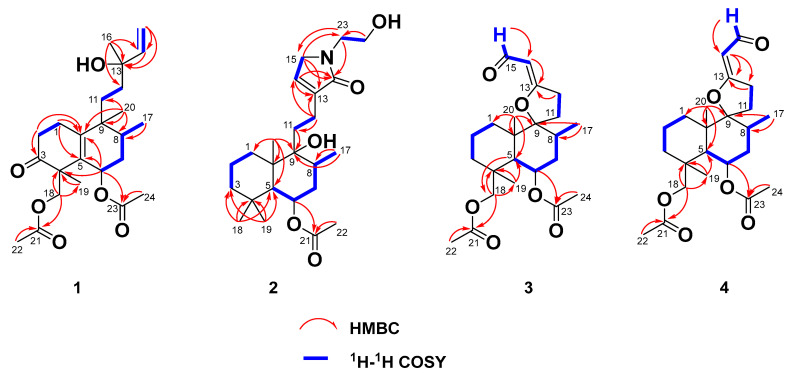
Key HMBC and ^1^H-^1^H COSY correlations of **1**–**4**.

**Figure 3:**
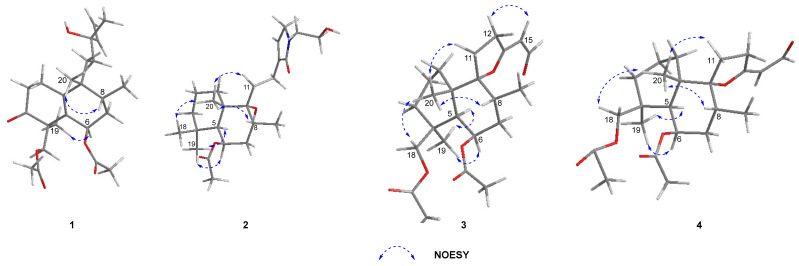
Key NOESY correlations of **1**–**4**.

**Figure 4:**
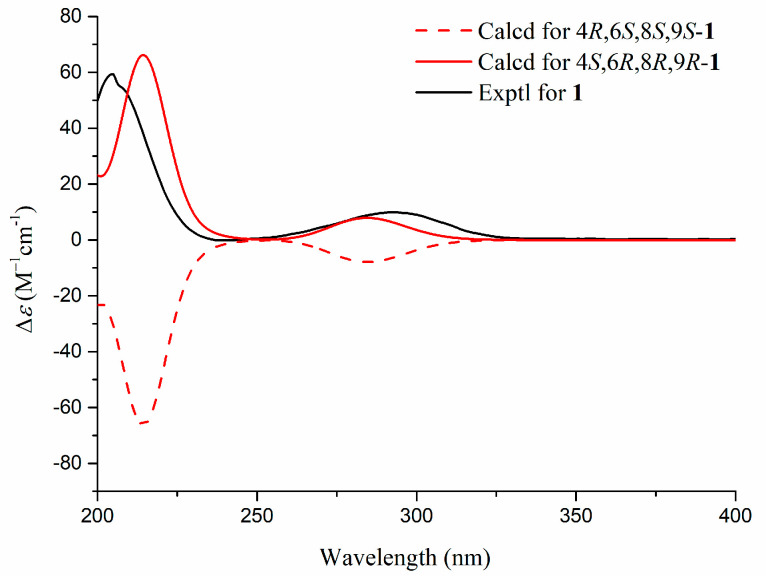
Comparison of the experimental and calculated ECD spectra of **1** (in MeOH).

**Figure 5:**
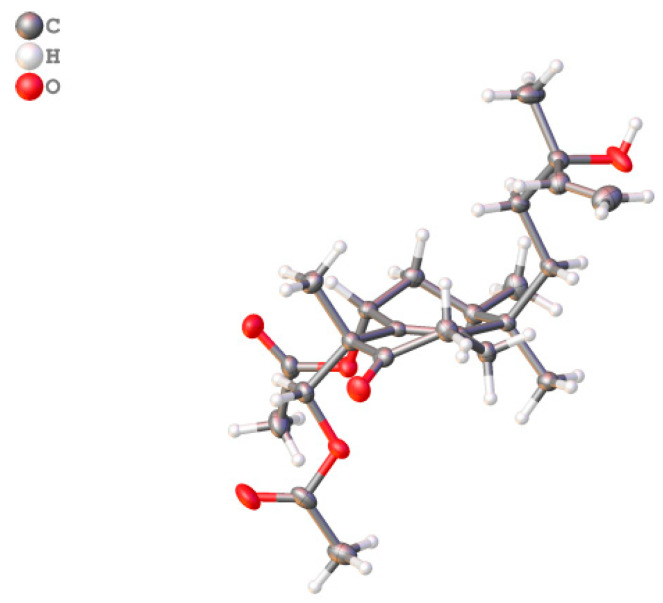
X-ray ORTEP drawing of compound **1** (Cu K*α*).

*Zeylleucapenoid A* (**1**) was obtained as colorless crystals. Analysis of the HR-ESI-MS spectrum showed it had a molecular formula of C_24_H_36_O_6_, indicating seven degrees of unsaturation. The ^1^H and ^13^C-NMR spectral data of **1** (Table 1) suggested that they were very similar to those of Leucasperone B [12], except for the absence of an oxygenated methine at (*δ*_H_ 4.15/*δ*_C_ 71.3, CH) in Leucasperone B, and the additional presence of a methylene at (*δ*_H_ 1.56/*δ*_C_ 30.0, CH_2_) in **1**. Based on this, compound **1** was considered an analogue of Leucasperone B. Further, the COSY cross-peak between H_2_-11/H_2_-12 confirmed that the methine of C-11 in Leucasperone B was replaced by a methylene in **1**, which was supported by the key HMBC correlations from H_2_-11 to C-8/C-10/C-13, and from both H-8 and H_3_-20 to C-10/C-11 (Figure 2). The partial relative configuration of **1** was determined via NOESY correlations showing cross-peaks between both H-6 and Me-17 with Me-19, and H-8 with Me-20, which indicated that H-6 and Me-19 were in the same orientation (Figure 3), while H-8 and Me-20 were in another orientation. Finally, a comparison of the experimental and calculated ECD spectra of **1** suggested that the absolute configuration of C-4/C-6/-C-8/C-9 in **1** was that of 4*S*,6*R*,8*R*,9*R*, which is attributed to the spectrum of the isomer (4*S*,6*R*,8*R*,9*R*) of **1** which showed a similar trend to the experimental curve (Figure 4). However, the absolute configuration of C-13 in **1** was still not assigned. Fortunately, a single crystal of **1** was attained and the absolute configuration of **1** (Figure 5) was clearly defined to be (4*S*,6*R*,8*R*,9*R*,13*S*) via X-ray diffraction analysis (CCDC no. 2225700), and named as zeylleucapenoid A.

**Figure 6:**
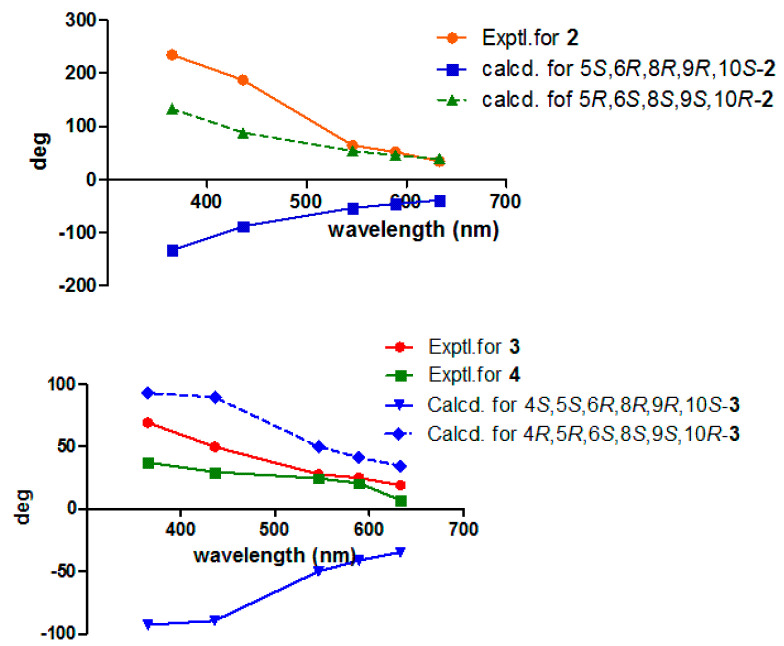
Experimental and calculated ORD spectra of **2**–**4**.

Compound **2** was purified as a white powder. Its molecular formula, C_24_H_39_NO_5_, was defined using the HR-ESI-MS spectrum with an ion peak at *m/z* 444.2725 [M+Na]^+^ (that calcd for C_24_H_39_NO_5_Na was 444.2726), corresponding to six degrees of unsaturation. The ^1^H NMR data of **2** (Table 1) showed characteristic resonances for an olefinic proton at *δ*_H_ 6.79 (H-14), three methines including an oxygenated proton at *δ*_H_ 5.23 (H-6), and five methyls at *δ*_H_ 0.85 (H-17), 0.88 (H-19), 0.94 (H-18), 1.19 (H-20) and 1.98 (H-22). The ^13^C NMR spectrum (Table 1) exhibited 24 carbon signals in total, including two amide/ester carbonyls at *δ*_C_ 170.8 (C-16)/169.9 (C-21), two sp^2^ carbons at *δ*_C_ 139.5 (C-13)/135.3 (C-14), four methyls at *δ*_C_ 33.4 (C-19)/23.5 (C-18)/21.6 (C-22)/16.0 (C-17), three sp^3^ methines at *δ*_C_ 69.5 (C-6)/46.7 (C-5)/31.1 (C-8), three sp^3^ quaternary carbons at *δ*_C_ 75.6 (C-9)/43.4 (C-10)/33.5 (C-4), and nine methylenes. These spectral data indicated that **2** was an analogue of vitexlactam A [13], except for the additional existence of an ethoxy moiety in **2**. Further analysis of 2D-NMR spectra of **2** confirmed the assignment above. In the COSY spectrum, the correlation between H_2_-23 and H_2_-24 was observable. In the HMBC spectrum, there were long-range correlations from H_2_-15 to C-23, and from H_2_-23 to C-15/C-16 (Figure 2), which suggested that the ethoxy moiety was connected to the nitrogen atom. The relative configuration of **2** was determined to be the same as that of vitexlactam A via an observation of NOESY correlations (Figure 3). In the NOESY spectrum, there were cross-peaks between Me-20 and H-8/H_2_-11/Me-18, indicating they were α-cofacial, while H-5/H-6/Me-19/9-OH were *β*-cofacial. The absolute configuration of **2** was determined to be 5*R*/6*S*/8*S*/9*S*/10*R* via a comparison of its specific rotation [*α*]^25^_D_ + 51.7 (*c* 2.1, MeOH) with that of vitexlactam A [*α*]^25^_D_−10.7 (*c* 0.42, CHCl_3_) [13], showing an opposite sign. Furthermore, a comparison of the calculated optical rotatory dispersion (ORD) spectrum of **2** tothe experimental one also supported that assignment, in which the calculated ORD spectrum of 5*R*,6*S*,8*S*,9*S*,10*R* of **2** agreed well with the experimental curve for **2** (Figure 6). Thus, **2** was elucidated as shown in Figure 1, and named zeylleucapenoid B.

Compound **3** was isolated as a colorless gum. Its molecular formula was determined to be C_23_H_34_O_6_ using HR-ESI-MS data at *m*/*z* 429.2256 (calcd as 429.2253 for C_23_H_34_O_6_Na). The ^1^H and ^13^C NMR data (Table 2) of **3** were found to be similar to those for 6*β*-acetoxy-9*α*,13-epoxy-16-norlabd-13*Z*-en-15-al, as previously reported from *L. zeylanica* by our team [11], except for an additional appearance of an acetyl group (*δ*_H_ 2.03/*δ*_C_ 20.5, *δ*_C_ 173.1) and an oxygenated methylene (*δ*_H_ 4.48/*δ*_C_ 67.6) in **3**, and the absence of a methyl (*δ*_H_ 1.05/*δ*_C_ 24.0) in 6*β*-acetoxy-9*α*,13-epoxy-16-norlabd-13*Z*-en-15-al. These data indicated that the methyl group in 6*β*-acetoxy-9*α*,13-epoxy-16-norlabd-13*Z*-en-15-al was oxygenated with an acetyl group in **3**. This was supported by the HMBC correlations from H-5 to C-18, H_2_-18 to C-3/C-4/C-19 and C-21, Me-19 to C-4/C-5 and C-18, and Me-22 to C-21. The (*Z*) configuration of the Δ^13(14)^ double bond was defined by the NOESY correlation of H_2_-12/H-14 (Figure 3). The relative configuration of **3** was assigned to be the same as that of 6*β*-acetoxy-9*α*,13-epoxy-16-norlabd-13*Z*-en-15-al, based on the NOESY correlations. The absolute configuration of **3** was established via a comparison of the experimental and calculated ORD spectra for it, which indicated that the calculated ORD spectrum (Figure 6) of 4*R*,5*R*,6*S*,8*S*,9*S*,10*R* of **3** agreed well with the experimental curve for **3**. Thus, the structure of zeylleucapenoid C (**3**) was established as shown in Figure 1.

Compound **4** was isolated as a colorless gum. Its molecular formula was determined to be C_23_H_34_O_6_ using HR-ESI-MS data at *m/z* 429.2260 (calcd to be 429.2253 for C_23_H_34_O_6_Na). The ^13^C NMR data (Table 2) closely resemble those of **3** except for a few deviations of chemical shifts from the signals for C-9 (Δ*δ*_C_ +1.8), C-11 (Δ*δ*_C_ −0.9), C-12 (Δ*δ*_C_ −0.8), C-13 (Δ*δ*_C_ −3.0), C-14 (Δ*δ*_C_ −1.0), and C-15 (Δ*δ*_C_ −2.5) in **4**. Analysis of its 2D NMR spectra showed that **4** is a stereoisomer of **3**. The main difference was the geometrical configuration of the Δ^13(14)^ double bond. The lack of NOESY correlations observed between H_2_-12 and H-14 and the ^3^*J*_12_,_14_ = 2.0 Hz (^3^*J*_12_,_14_ = 0 Hz in **3**) also verified that the Δ^13(14)^ double bond was *E*-formed. Because the experimental ORD spectrum (Figure 6) of **4** was similar to that of **3**, the absolute configuration of **4** was determined to be (4*R*,5*R*,6*S*,8*S*,9*S*,10*R*), and named zeylleucapenoid D.

### 2.2. Anti-Inflammatory Activity

**Figure 7:**
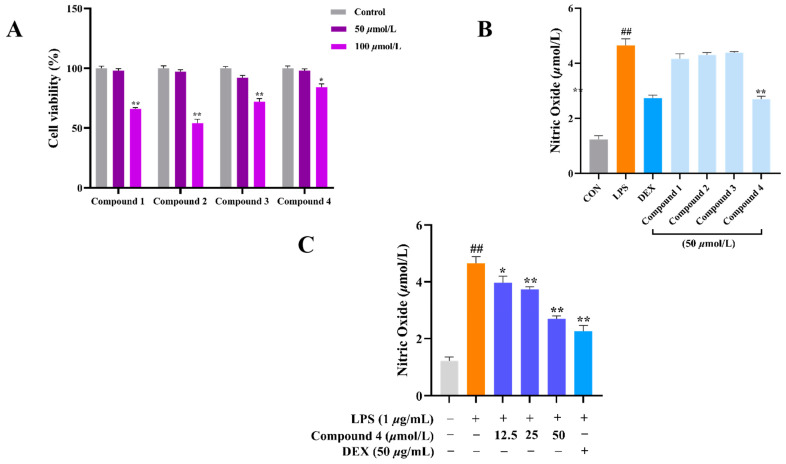
The effects of the administered dose of compounds **1**–**4** on cell viability and NO levels. (**A**) Cell viability of compounds **1**–**4** at the dose of 50, 100 μM. * *p* < 0.05 and ** *p* < 0.01 vs. Con. *n* ≥ 3. (**B**) The levels of NO in LPS inducing RAW264.7 macrophages at a concentration of 50 μM for samples. ^##^ *p* < 0.01 vs. Con, ** *p* < 0.01 vs. LPS. *n* ≥ 3. (**C**) Compound **4** reducing LPS-induced RAW264.7 macrophages’ NO levels. ^##^ *p* < 0.01 vs. Con, * *p* < 0.05, and ** *p* < 0.01 vs. LPS. *n* ≥ 3.

**Figure 8:**
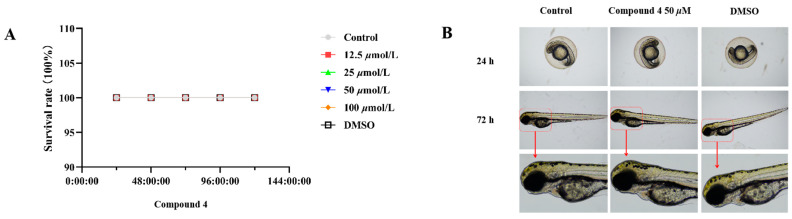
The toxic effect of compound **4** on zebrafish. (**A**) The toxicity of compound **4** was used to test with the zebrafish model. At 1–5 dpf, zebrafish embryos were subjected to different compound concentrations (12.5, 25, 50, and 100 μM). The number of dead embryos were recorded every day. (**B**) Effect of compound **4** on the development and morphology of zebrafish embryos.

**Figure 9:**
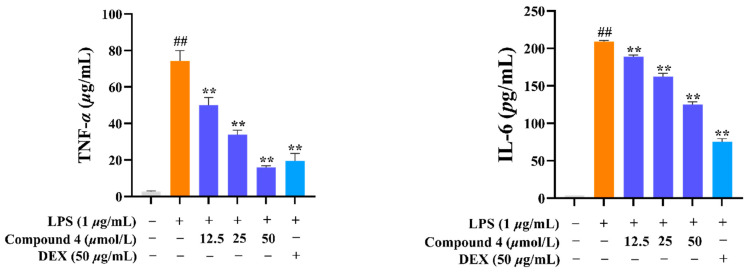
Impact of compound **4** on cytokine secretion in LPS-treated RAW264.7 cells. Cell pre-treatment was performed for 1 h using different concentrations compound **4** concentrations (12.5, 25, and 50 μM) followed by LPS (1 μg/mL) treatment for 24 h. Supernatants of the cell cultures were obtained and used to determine IL-6 and TNF-*α* levels via ELISA. ^##^ *p* < 0.01 vs. Con, and ** *p* < 0.01 vs. LPS. *n* ≥ 3.

**Figure 10:**
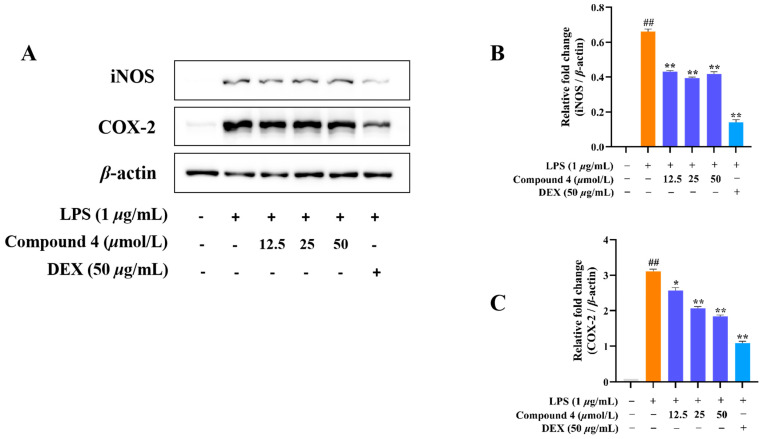
Inhibition of LPS-induced iNOS and COX-2 gene expression in RAW264.7 cells for compound **4**. RAW264.7 cells were preincubated using compound **4** for 1 h, followed by being co-treated with LPS for 24 h, and analyzed via Western blotting. (**A**) Western blotting; (**B**) iNOS expression; (**C**) COX-2 expression. Data are expressed as mean ± SD (*n* ≥ 3). ^##^ *p* < 0.01 vs. Con, * *p* < 0.05, and ** *p* < 0.01 vs. LPS. *n* ≥ 3.

Considering the traditional anti-inflammatory efficacy of *L. zeylanica*, compounds **1**–**4** were examined for their ability to inhibit nitric oxide (NO) production [14]. Prior to the bioassay, the in vitro cytotoxic effects against cell viability were detected using the MTT method, and compounds **1**–**4** showed no cytotoxic activity with CC_50_ values of >100 μM. At non-cytotoxic concentrations, compound **4** exhibited significant effects on reducing the LPS-induced NO production with an IC_50_ value of 38.45 μM in RAW264.7 macrophages, while the positive control dexamethasone showed an IC_50_ value of 79.34 μM (Figure 7). Meanwhile, the zebrafish embryo toxicity test was thought to be suitable for the evaluation of the toxic property of drug candidates. Herein, active compound **4** was evaluated for toxicity activity using the zebrafish embryo model, which indicated that **4** showed nontoxic activity at the concentrations of 12.5, 50 and 100 μM, respectively (Figure 8). A subsequent ELISA assay uncovered that **4** could strongly suppress the secretion of LPS-induced TNF-*α* and IL-6 cytokines in a dose-dependent manner for RAW264.7 macrophages (Figure 9). In order to understand the possible anti-inflammatory mechanism, the effects of **4** on iNOS and COX-2 protein expression levels were examined via Western blotting, which indicated that compound **4** dose-dependently attenuated the levels of the inflammatory mediators iNOS and COX-2 (Figure 10). Based on the above, these data disclosed that compound **4** played an important role through the downregulation of pro-inflammatory enzyme expression, leading to an anti-inflammatory effect. To the best of our knowledge, there are a few reports of halimane-type diterpenoids without anti-inflammatory activity against NO release [15]. Combining our results of anti-inflammatory activity for **1**–**4**, it may be concluded that the substituent with a spiro-ring unit at C-9 was a potentially functionalized group. In addition, our discovery of non-toxicity against zebrafish embryos and binding with iNOS and COX-2 for **4** will promote the yield of lead compounds via further structural prioritization.

### 2.3. Predicted Binding Modes of Compound ***4*** and Both iNOS and COX-2 Using Molecular Docking Analysis

**Figure 11:**
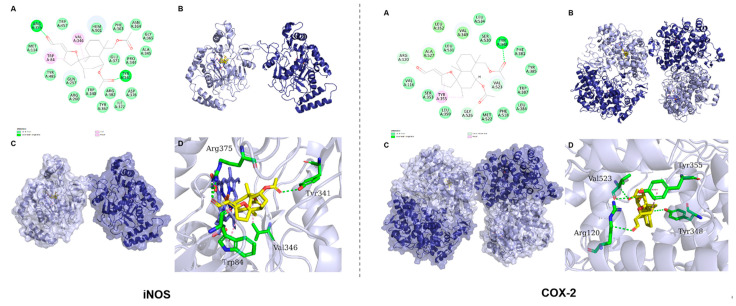
The binding mode of 4 with iNOS and COX-2. (**A**) The 2D binding mode of 4 with iNOS and COX-2. The green and light-green dash lines are depicted as hydrogen bond. The pink dash line is depicted as hydrophobic. (**B**,**C**) The 3D surface and cartoon binding mode of 4 with iNOS and COX-2. (**D**) The detailed 3D binding mode of 4 with iNOS and COX-2. Compound is depicted as yellow sticks, the surrounding residues in the binding pockets are depicted as green sticks, the backbone of the receptor is depicted as lightblue and deepblue cartoon. The hydrogen and hydrophobic bonds are depicted as green and pink dashed lines.

To further recognize the possible binding modes of anti-inflammatory activity for **4**, a molecular docking study was performed on **4** and both iNOS and COX-2 proteins. The result showed that **4** was well-accommodated in the binding pocket of iNOS, primarily interacted with Tyr341 and Arg375 residues through stable hydrogen bonds, and interacted with residues Trp84 and Val346 through hydrophobic bonds (Figure 11). Meanwhile, in the binding pocket of COX-2, compound **4** mainly formed stable hydrogen bonds with Tyr348, Val523 and Arg120 residues, and formed hydrophobic bonds with residues Val523 and Tyr355 (Figure 11). The lower binding energies of −5.862 and −6.722 kcal/mol also provided reliable evidence to confirm their strong affinity (Table 3). Therefore, the molecular docking analysis provided a perspective on the potential targets for **4**, which will be helpful for discovering the specific binding site in a follow-up experiment.

## 3. Materials and Methods

### 3.1. General Experimental Procedures

The optical rotation value was tested through a JASCO P-1020 digital polarimeter (JASCO, Tokyo, Japan), while the acquisition of ECD spectra was carried out using a Jasco J-815 (JASCO, Tokyo, Japan) circular dichroism spectrometer at room temperature. Briefly, 1D and 2D NMR data were recorded on a Bruker AV (Bruker Corporation, Basel, Switzerland) spectrometer (400 MHz for ^1^H and 100 MHz for ^13^C), while TMS was used as an internal reference. The acquisition of HRESIMS data was carried out via a Q-TOF Ultima Global GAA076 LC (Billerica, MA, USA) mass spectrometer. Semi-preparative HPLC was carried out on an Agilent 1260 LC (Agilent Corporation, Santa Clara, CA, USA) infinity series, by loading an Agilent Eclipse XDB-C_18_ column (9.4 × 250 mm, 5 μm, Agilent Corporation, Santa Clara, CA, USA), using a DAD-UV detector. Silica gel (Qing Dao Hai Yang Chemical Group Co., Qing dao, China; 100–200, 200–300 mesh) was employed in column chromatography (CC). Thin-layer chromatography (TLC) (Yan Tai Zi Fu Chemical Group Co., Yan Tai, China, G60, F-254) was used to monitor the separation of samples. Anti-inflammatory activity was evaluated using a Microplate spectrophotometer (Bio-Rad, California, USA) as a template reader.

### 3.2. Plant Material

The aerial parts of *Leucas zeylanica* (Lamiaceae) were collected from Changjiang city, Hainan province of China, in July 2020, and were authenticated by Professor Yu-Kai Chen (School of Hainan Normal University, Changjiang, Hainan, China). The specimens (no. C20-L02) were deposited at the Key Laboratory of Tropical Medicinal Resource Chemistry of Ministry of Education, Hainan Normal University (Haikou, Hainan, China).

### 3.3. Extraction and Isolation

The aerial parts of *L. zeylanica* (10.0 kg) were extracted with 95% EtOH (3 × 25 L). A dark-brown crude extract (1.1 kg) was obtained after concentration in vacuo to remove most of the EtOH. The crude extract was suspended in distilled water and partitioned with PE (60–90) (3.0 × 1.0 L), EtOAc (3.0 × 1.0 L) and *n*-BuOH (3.0 × 1.0 L), yielding 90, 295 and 354 g of residues, respectively.

The PE-soluble fraction (87 g) was subjected to silica-gel column chromatography (CC) (100–200 mesh) with gradient elution (petroleum ether/ethyl acetate, 100:0, 90:10, 80:20, 70:30, 60:40, and 0:100; *v*/*v*, 6 L each) to obtain four major fractions (Fr. P1-P4).

The Fr. P-3 (15 g) part was separated by a silica-gel column and eluted with gradient mixtures of petroleum ether–acetone (from 5:1 to 1:1) to obtain fractions (Fr. P3-1-P3-5). Fraction Fr. P3-3 (6 g) was separated on Sephadex LH-20 (Pharmacia, Beijing, China) (CHCl_3_: MeOH, 1:1) and a RP-C_18_ silica-gel column (Qing Dao Hai Yang Chemical Group Co., Qingdao, China) (MeOH/H_2_O, from 70% to 100%) to obtain fractions of Fr. P33-1-P33-6 on the basis of TLC analysis. Fraction Fr. P3.3-3 (160 mg) was purified via semi-preparative HPLC with MeOH/H_2_O (60:40 *v*/*v*) as an eluent to obtain compound **1** (12 mg).

The EtOAc-soluble fraction (290 g) was subjected to silica gel column chromatography (CC) (100–200 mesh) with gradient elution (petroleum ether/ethyl acetate and chloroform/methanol, 100:0, 90:10, 80:20, 70:30, 60:40, and 0:100; *v*/*v*, 8 L each) to obtain six major fractions (Fr. E1-E2).

Fr. E-3 (24.2 g) was subjected to an ODS column and eluted with MeOH/H_2_O (from 10:90 to 100:0 *v*/*v*), obtaining seven sub-fractions (Fr. E3-1-Fr. E3-7). Fr. E3-3 (1.7 g) was purified on a silica-gel column (200-300 mesh, petroleum ether–EtOAc, 100:0, 90:10, 80:20, 70:30, 60:40, and 0:100 *v*/*v*) to yield five additional fractions (Fr. E33-1-Fr. E33-5). Fr. E33-3 (336 mg) was decolorized using a silicone column and eluted via gradient elution (MeOH-H_2_O, from 80:20 to 100:0 *v*/*v*), to yield three subfractions (Fr. E333-1-Fr. E333-3). Compounds **3** (2.1 mg) and **4** (5.6 mg) were obtained from Fr. E333-2 (87 mg) via HPLC (MeOH-H_2_O, 61:39 *v*/*v*).

Fr. E-5 (1.2 g) was subjected to silica-gel column chromatography (CC) (200–300 mesh) using CHCl_3_-MeOH (100:0, 90:10, 80:20, 70:30, 60:40, and 0:100; *v/v*) with gradient elution to retrieve five fractions (Fr. E5-1-Fr. E5-5). Fr. E5-3 (540 mg) was subjected to an ODS column eluting with MeOH/H_2_O (from 20:80 to 100:0 *v*/*v*) to obtain four subfractions (Fr. E53-1-Fr. E53-4). Fr. E53-3 (118 mg) was further purified via HPLC (MeOH-H_2_O, 68:32) to obtain compound **2** (7.8 mg).

#### 3.3.1. Zeylleucapenoid A (**1**)

Colorless block crystals; [*α*]^25^_D_ **+**11.43 (*c* 0.14, MeOH); mp 156.6–157.9 °C; UV (MeOH) *λ*_max_ (log *ε*) 220 (3.91), 266 (3.02), 275 (2.95) nm; CD (*c* 0.0005, MeOH) *λ*_max_ (Δ*ε*) 205 (+59.40), 293 (+9.90) nm; ^1^H NMR (400 MHz, CD_3_OD) and ^13^C NMR (100 MHz, CD_3_OD), see Table 1; HR-ESI-MS *m*/*z* 443.2415 (calcd as 444.2410 for C_24_H_36_O_6_Na).

#### 3.3.2. Zeylleucapenoid B (**2**)

White powder; [*α*]^25^_D_ +51.7 (*c* 2.1, MeOH); UV (MeOH) *λ*_max_ (log *ε*) 222 (3.99), 306 (3.02), 318 (2.93) nm; ^1^H NMR (400 MHz, CD_3_OD) and ^13^C NMR (100 MHz, CD_3_OD), see Table 1; HR-ESI-MS *m/z* 444.2725 (calcd as 444.2726 for C_24_H_39_NO_5_Na).

#### 3.3.3. Zeylleucapenoid C (**3**)

Colorless gum; [*α*]^25^_D_ +24.84 (*c* 1.0, MeOH); UV (MeOH) *λ*_max_ (log *ε*) 220 (3.90), 266 (3.25) nm; ^1^H NMR (400 MHz, CD_3_OD) and ^13^C NMR (100 MHz, CD_3_OD), see Table 2; HR-ESI-MS *m*/*z* 429.2256 (calcd as 429.2253 for C_23_H_34_O_6_Na).

#### 3.3.4. Zeylleucapenoid D (**4**)

Colorless gum; [*α*]^25^_D_ +20.53 (*c* 1.5, MeOH); UV (MeOH) *λ*_max_ (log *ε*) 219 (3.95), 267 (3.69) nm; ^1^H NMR (400 MHz, CD_3_OD) and ^13^C NMR (100 MHz, CD_3_OD), see Table 2; HR-ESI-MS *m/z* 429.2260 (calcd as 429.2253 for C_23_H_34_O_6_Na).

### 3.4. X-ray Crystallographic Analysis

Crystals of compound **1** were obtained from MeOH at room temperature. Single-crystal X-ray diffraction data were collected on a Rigaku, Oxford, diffractometer (Oxford Diffraction Ltd.: Abingdon, England, UK) with Cu K*α* radiation (*λ* = 1.54184 Å) at 100.00(10) K, respectively. Using the direct methods (ShelXS) and refinement with the ShelXL program, structure determination and refinement were performed. Crystallographic data of compound **1** were deposited in the Cambridge Crystallographic Data Centre (CCDC numbers: 2225700 for **1**). The data can be obtained free of charge from the Cambridge Crystallographic Data Centre (https://www.ccdc.cam.ac.uk/, accessed on 11 December 2022).

Crystal data of compound **1**: C_24_H_36_O_6_, *M_r_* = 420.53; colorless block crystals from CH_3_OH; crystal size = 0.25 × 0.16 × 0.14 mm^3^; T = 100.00(10) K; space group P2_1_; monoclinic, *a* = 9.33510 (10) Å, *b* = 9.69210 (10) Å, *c* = 13.5574 (2) Å, *α* = 90°, *β* = 107.5540(10), *γ* = 90°, *V* = 1169.51(3) Å^3^, *Z* = 2, *D*_calc_ = 1.194 g/cm^3^, *F* (000) = 456.0, and μ (Cu K*α*) = 0.685 mm^−1^. Independent reflections: 4731 unique (*R*_int_ = 0.0227, *R*_sigma_ = 0.0194). The final *R*_1_ was 0.0288 and *wR*_2_ was 0.0751 [I ≥ 2*σ* (I)] (all data). Flack parameter = 0.00(5). CCDC no. 2225700 (Table S1 in the Supporting Information).

### 3.5. Anti-Inflammatory Activity

#### 3.5.1. NO Measurement

All isolated compounds were evaluated for their inhibition of nitric oxide (NO) production in RAW264.7 cells activated by lipopoly saccharide (LPS) using the Griess assay with dexamethasone (DEX) as a positive control [16,17]. RAW 264.7 cells were seeded in 96-well plates at a density of 2 × 10^5^ cells/mL. After 12 h of incubation, the cells were pre-treated with the compounds (50 μM) and DEX (50 μg/mL) for 1 h and following additional LPS (1 μg/mL) treatment for 24 h at 37 °C. After 24 h, the quantity of NO accumulated in the culture medium was measured. Briefly, to the cell culture medium (50 µL) was added an equivalent volume of the Griess reagent. The absorbance was measured using a microplate reader at 540 nm wavelength.

#### 3.5.2. The MTT Assay

Briefly, RAW264.7 cells were seeded in 96-well plates at a density of 1 × 10^5^ cells/mL. Incubation was performed for 12 h after which the compounds (50 μM) were used to treat the cells for 24 h. Subsequently, 20 μL of the MTT stock solution (5 mg/mL) was added to the wells. After 4 h incubation, the supernatants were aspirated. The formazan crystals in each well were dissolved in DMSO (150 µL), and the absorbance was measured at a wavelength of 570 nm using a microplate reader. The data were expressed as mean percentages of the viable cells compared to the respective control.

#### 3.5.3. Zebrafish Maintenance

Adult wild-type zebrafish (Danio rerio) were raised at a standard facility, which allows the control of stationary light and temperature. The zebrafish were treated with a light/dark photoperiod in 14:10 h cycles, and fed live brine shrimp 2 times a day. Further, the embryos were produced from the spawning of adult fish using a hatch box, and the incubation process from embryo to larvae was maintained at 28 °C. The larvae were collected and used for the toxic experiments. All the zebrafish procedures were approved by the Institutional Animal Care and Committee of Hainan Normal University.

#### 3.5.4. Toxic Effects in Zebrafish

Although the anti-inflammatory activity of diterpenoids in zebrafish models has been reported previously [18,19,20], it is still not very common. Compound **4** was evaluated for zebrafish larval toxicity studies. Four hours post-fertilization (hpf) larvae were placed in 6-well plates at a count of 10 fish/well and compounds were added in the fish water at 4 different concentrations (12.5, 25, 50, and 100 µM). The EVOS digital microscope (4×) was used to detect toxic activity for the larval zebrafish up to 120 hpe (hours post-exposure). Prior to the test, larval zebrafish were checked for their viability, where a lack of heartbeat was considered death (acute toxic dose). Other indications of toxicity involved swim position, and morphological deficits such as malformations, larval length, tail curvature, and swim bladder inflation level.

#### 3.5.5. ELISA Assay

Cytokine levels were quantified using ELISA kits in accordance with the manufacturer’s protocol [21,22]. After pretreatment with compound **4** (12.5, 25 and 50 μM) and DEX (50 μg/mL) for 1 h, cells were incubated with compound **4** and LPS for an additional 24 h, and cell culture supernatants were collected. The expression levels of IL-6 and TNF-*α* in the culture medium were assessed by measuring the absorbance at 450 nm using a microplate reader.

#### 3.5.6. Western Blot Analysis

RAW264.7 cells were seeded at a density of 1 × 10^6^ cells/well in 6-well plates for 24 h [23,24]. Cells were then pretreated with compound **4** for 1 h and stimulated with LPS (1 µg/mL). After 24 h of continuous incubation, cells were washed twice with cold PBS and collected. Cells were lysed with a lysis buffer containing a freshly added protease inhibitor cocktail and phenylmethyl sulfonylfluoride. The lysate was then centrifuged at 12,000 rpm for 10 min and the supernatant was collected to obtain the total protein concentration. Protein concentrations were determined using BCA Protein Assay Kit (Beyotime Biotechnology, Shanghai, China). Equal amounts of protein were separated via SDS-PAGE(Beijing Liuyi Biotechnology Co., Ltd., Beijing, China) gel electrophoresis and transferred to polyvinylidene difluoride membranes. Membranes were blocked with 5% skimmed milk for 2 h at room temperature and then the membranes were further incubated with a primary antibody (iNOS and COX-2) at 4 °C overnight followed by incubation with a horseradish peroxidase-conjugated secondary antibody. Finally, protein blots were visualized using an ECL detection kit (Beyotime Biotechnology). *β*-actin was used as an internal reference. Each band was quantified using Image J software.

#### 3.5.7. Molecular Docking Studies

Molecular docking was conducted in AutoDock using the hybrid Lamarckian Genetic Algorithm (LGA) [25,26]. The 3D structure of iNOS (PDB:3E6T) and COX-2 (PDB:1PXX) was downloaded from RCSB PDB (https://www.rcsb.org/, accessed on 5 December 2022). The 3D structure of **4** was drawn in ChemDraw (https://www.chemdraw.com.cn/, accessed on 5 December 2022) as ligands. The protein and ligand were converted to a PDBQT format using AutoDockTools. The ligands were set to flexible; the receptor was set to rigid. The conformation with the lowest binding free energy was finally identified as the best probable binding mode. Water molecules and the original ligand of the receptor were manually removed using PyMol software. Prepare_ligand4.py and prepare_recptor4.py scripts from AutoDockTools 1.5.6 were used to prepare the initial files of ligands including adding charges and hydrogen atoms.

## 4. Conclusions

Chemical investigations of the 95% EtOH extract of *L. zeylanica* allowed the obtention of four undescribed highly oxygenated halimane-type and labdane-type diterpenoids (**1**–**4**). The absolute configuration of the new compound, **1**, was determined using theoretical ECD calculations and single-crystal diffraction. The absolute configuration of new compounds **2**–**4** was determined using theoretical ORD calculations. Among them, compound **4** showed significant anti-inflammatory activity against LPS-induced NO, TNF-α and IL-6 production, and the inhibition of iNOS and COX-2 protein expression levels. The molecular docking analysis indicated that **4** had a strong affinity with both iNOS and COX-2 through hydrogen and hydrophobic bond interactions with a few amino acid residues. These results were significative of the discovery of anti-inflammatory target and lead compounds for the treatment of inflammation-linked diseases.

## Figures and Tables

**Table 1:** ^1^H and ^13^C NMR spectral data of compounds **1**–**2**.

Position	1 ^a^		2 ^b^	
	*δ*_H_, Mult, (*J* in Hz)	*δ*_C_	*δ*_H_, Mult, (*J* in Hz)	*δ*_C_
1	2.47, m; 2.59, m	24.2	1.34, m; 1.59, m	35.9
2	2.51, m	38.4	1.41, m 1.55, m	18.4
3	-	214.7	1.10, m; 1.24, m	44.5
4	-	52.1	-	33.5
5	-	130.3	1.68, d, (2.4)	46.7
6	5.43, t, (2.8)	68.5	5.23, dd, (5.6, 2.8)	69.5
7	1.71, m	36.0	1.35, m 1.62, m	32.2
8	1.85, m	34.5	2.02, m	31.1
9	-	42.6	-	75.6
10	-	149.8	-	43.4
11	1.56, m	30.0	1.37, m 1.57, m	33.0
12	1.21, m 1.63, m	40.4	2.23, t, (7.2)	22.5
13	-	73.9	-	139.5
14	5.87, dd, (17.6, 10.8)	146.2	6.79, s	135.3
15	5.05, dd, (10.8, 2.0) 5.20, dd, (17.6, 2.0)	112.4	3.96, s	51.5
16	1.15, s	27.7	-	170.8
17	1.04, d, (7.2)	16.6	0.85, d, (6.8)	16.0
18	4.05, d, (10.8) 4.20, d, (10.8)	69.5	0.94, s	23.5
19	1.18, s	20.1	0.88, s	33.4
20	1.24, s	27.5	1.19, s	19.0
21	-	172.2	-	169.9
22	1.98, s	20.7	1.98, s	21.6
23	-	172.4	3.38, t, (5.8)	43.5
24	2.00, s	21.5	3.50, t, (5.8)	59.5

^a^ measured in CD_3_OD at 400 MHz; ^b^ measured in DMSO-*d*_6_ at 400 MHz.

**Table 2:** ^1^H and ^13^C NMR spectral data of compounds **3**–**4**.

Position	3 ^a^		4 ^a^	
	*δ*_H_, Mult, (*J* in Hz)	*δ*_C_	*δ*_H_, Mult, (*J* in Hz)	*δ*_C_
1	1.25, m; 1.51, m	33.7	1.21, m; 1.48, m	33.7
2	1.55, m; 1.72, m	18.9	1.66, m; 1.53, m	18.9
3	1.89, m; 1.75, m	37.4	1.87, m	37.3
4	-	39.3	-	39.2
5	1.86, d, (2.4)	51.7	1.81, d, (2.4)	51.4
6	5.51, dd, (5.6, 2.8)	70.7	5.48, q, (2.4)	70.7
7	1.75, m; 1.00, m	37.1	1.70, m; 0.97, m	37.0
8	2.24, m	33.2	2.27, m	33.1
9	-	103.3	-	101.5
10	-	44.2	-	43.9
11	2.31, m; 1.98, m	25.8	2.36, m; 2.06, m	26.7
12	2.96, m	30.1	3.20, m	30.9
13	-	183.6	-	186.6
14	5.09, d, (8.8)	100.7	5.55, dt, (8.0, 2.0)	101.7
15	9.86, d, (8.8)	190.7	9.49, d, (8.0)	193.2
17	0.88, d, (6.4)	15.8	0.85, d, (6.4)	15.7
18	4.13, dd, (11.2, 1.6) 4.48, d, (11.2)	67.6	4.41, dd, (11.2, 1.6) 4.47, d, (11.2)	67.6
19	1.05, s	27.4	1.03, s	27.4
20	1.33, s	20.7	1.34, s	20.7
21	-	173.1	-	173.1
22	2.03, s	20.5	2.03, s	20.6
23	-	172.0	-	172.0
24	2.10, s	21.8	2.09, s	21.8

^a^ measured in CD_3_OD at 400 MHz.

**Table 3:** Logarithms of free binding energies (FBE, kcal/mol) between compound **4** and the active cavities of both iNOS (PDB code, 3E6T) and COX-2 (PDB code, 1PXX) via targeting residues.

Compounds	Protein	−Log(FBE)	Targeting Residues			
**4**	iNOS	−5.862	Tyr341	Arg375	Trp84	Val346
	COX-2	−6.722	Tyr348	Val523	Arg120	Tyr355

## Data Availability

The data of the article can be obtained from the authors.

## Supplementary Materials

The following supporting information can be downloaded at https://www.mdpi.com/article/10.3390/molecules28114472/s1. Table S1: Crystal data and structure refinement for compound **1** using autored. Table S2: Fractional atomic coordinates (×10^4^) and equivalent isotropic displacement parameters (Å2 × 10^3^) for compound **1** using autored. Ueq is defined as 1/3 of the trace of the orthogonalised UIJ tensor. Table S3: Anisotropic displacement parameters (Å2 × 103) for LE35113_A_autored. The anisotropic displacement factor exponent takes the form −2π2 [h2a × 2U11 + 2hka × b × U12 + …]. Table S4: Bond lengths for compound **1** determined using autored. Table S5: Bond angles for compound 1 determined using autored. Table S6: Hydrogen bonds for compound **1** determined using autored. Table S7: Torsion angles for compound 1 determined using autored. Table S8: Hydrogen Atom coordinates (Å × 10^4^) and isotropic displacement parameters (Å2 × 10^3^) for compound **1** determined using autored. Computational Section—Table S9: Energies of the dominative conformers of compound **1**. Table S10: Energies of the dominative conformers of compounds **2** and **3**. Table S11: Calculated and measured OR values of compounds **2** and **3** at different wavelengths. Table S12 The docking pockets. Figure S1: UV Spectrum of compound **1**. Figure S2: (+)-HRMS(ESI) spectroscopic data of compound **1**. Figure S3: ^1^H NMR Spectrum of compound **1** in CD_3_OD. Figure S4: ^13^C NMR spectrum of compound **1** in CD_3_OD. Figure S5: DEPT spectrum of compound **1** in CD_3_OD. Figure S6: HSQC spectrum of compound **1** in CD_3_OD. Figure S7: ^1^H-^1^H COSY spectrum of compound **1** in CD_3_OD. Figure S8: HMBC spectrum of compound **1** in CD_3_OD. Figure S9: NOESY spectrum of compound **1** in CD_3_OD. Figure S10: UV spectrum of compound **2**. Figure S11: (+)-HRMS(ESI) spectroscopic data of compound 2. Figure S12: ^1^H NMR spectrum of compound **2** in DMSO-*d*_6_. Figure S13: ^13^C NMR spectrum of compound **2** in DMSO-*d*_6_. Figure S14: DEPT spectrum of compound **2** in DMSO-*d*_6_. Figure S15: HSQC spectrum of compound **2** in DMSO-*d*_6_. Figure S16: ^1^H-^1^H COSY spectrum of compound **2** in DMSO-*d*_6_. Figure S17: HMBC spectrum of compound **2** in DMSO-*d*_6_. Figure S18: NOESY spectrum of compound **2** in DMSO-*d*_6_. Figure S19: UV spectrum of compound **3**. Figure S20: (+)-HRMS(ESI) spectroscopic data of compound **3**. Figure S21: ^1^H NMR spectrum of compound **3** in CD_3_OD. Figure S22: ^13^C NMR spectrum of compound **3** in CD_3_OD. Figure S23: DEPT spectrum of compound **3** in CD_3_OD. Figure S24: HSQC spectrum of compound **3** in CD_3_OD. Figure S25: ^1^H-^1^H COSY spectrum of compound **3** in CD_3_OD. Figure S26: HMBC spectrum of compound **3** in CD_3_OD. Figure S27: NOESY spectrum of compound **3** in CD_3_OD. Figure S28: UV spectrum of compound **4**. Figure S29v: (+)-HRMS(ESI) spectroscopic data of compound **4**. Figure S30: ^1^H NMR spectrum of compound **4** in CD_3_OD. Figure S31: ^13^C NMR spectrum of compound **4** in CD_3_OD. Figure S32: DEPT spectrum of compound **4** in CD_3_OD. Figure S33: HSQC spectrum of compound **4** in CD_3_OD. Figure S34: ^1^H-^1^H COSY spectrum of compound **4** in CD_3_OD. Figure S35: HMBC spectrum of compound **4** in CD_3_OD. Figure S36: NOESY spectrum of compound **4** in CD_3_OD.

## Sample Availability

Not available.
