# Supplementary material for: Zeylleucapenoids A–D, Highly Oxygenated Diterpenoids with Anti-Inflammatory Activity from Leucas zeylanica (L.) R. Br
Source: Molecules. 2023 May 31;28(11):4472. doi: 10.3390/molecules28114472 (PMC10254181; doi:10.3390/molecules28114472)
Supplement: Supplementary file 1 [file molecules-28-04472-s001.zip › molecules-2372056-supplementary.pdf]

SUPPLEMENTARY MATERIAL

Zeylleucapenoids A–D, Highly Oxygenated Diterpenoids with Anti-Inflammatory Activity from *Leucas zeylanica* (L.) R. Br.

Ting Zhao [1,2,†], Xuan Zhang [1,2,†], Xu-Hua Nong [1,2], Xue-Ming Zhou [1,2], Ru-Ru Chai [1,2], Xiao-Bao Li [1,2,*] and Guang-Ying Chen [1,2,*]

1 Key Laboratory of Tropical Medicinal Resource Chemistry of Ministry of Education Hainan Normal University, Haikou 571158, China; zhaoting19930812@126.com (T.Z.); zxuan0328@163.com (X.Z.); nongxuhua4883@163.com (X.-H.N.); xueming2009211@126.com (X.-M.Z.); 13119000891@163.com (R.-R.C.)

2 Key Laboratory of Tropical Medicinal Plant Chemistry of Hainan Province, College of Chemistry and Chemical Engineering, Hainan Normal University, Haikou 571158, China

* Correspondence: lixiaobao0797@163.com (X.-B.L.); chgying123@163.com (G.-Y.C.)

† These authors contributed equally to this work.

Abstract

Four previously undescribed highly oxygenated diterpenoids (**1**–**4**), zeylleucapenoids A-D, characterized by halimane and labdane skeletons, were isolated from the aerial parts of *Leucas zeylanica*. Their structures were elucidated primarily by NMR experiments. The absolute configuration of **1** was established by X-ray crystallographic analysis, whereas those for **2-4** were assigned by quantum-chemical calculations. Zeylleucapenoid D, showed significant anti-inflammatory activity and obviously inhibited pro-inflammatory cytokines TNF-*α* and IL-6 in a dose-dependent manner with nontoxic activity for zebrafish embryo. Furthermore, by regulation of the expression of inducible nitric oxide synthase (iNOS) and cyclooxygenase-2 (COX-2) proteins as well as their binding interactions with the two proteins via Western blotting and molecular docking to explore the possible mechanism.

Keywords

Leucas zeylanica; Highly oxygenated; Diterpenoids; Anti-inflammatory activity; Molecular docking; Zebrafish model

The list of Content

No.	Content	Page
1	**Table S1** Crystal data and structure refinement for compound **1** using autored.	1
2	**Table S2** Fractional atomic coordinates (×10^4) and equivalent isotropic displacement parameters (Å2×10^3) for compound **1** using autored. Ueq is defined as 1/3 of of the trace of the orthogonalised UIJ tensor.	2
3	**Table S3** Anisotropic displacement parameters (Å2×10^3) for LE35113_A_autored. The anisotropic displacement factor exponent takes the form: -2π2[h2a*2U11+2hka*b*U12+…].	3
4	**Table S4** Bond Lengths for compound **1** determined using autored.	4
5	**Table S5** Bond angles for compound **1** determined using autored.	5
6	**Table S6** Hydrogen Bonds for compound **1** determined using autored.	6
7	**Table S7** Torsion angles for compound **1** determined using autored.	6
8	**Table S8** Hydrogen atom coordinates (Å×10^4) and isotropic displacement parameters (Å2×10^3) for compound **1** determined using autored.	7
9	**Computational Section**	8
10	**Table S9** Energies of the dominative conformers of compound **1**.	8
11	**Table S10** Energies of the dominative conformers of compounds **2** and **3.**	9
12	**Table S11** Calculated and measured OR values of compounds **2** and **3** at different wavelengths.	10
13	**Table S12** The docking pockets.	11
14	**Figure S1** The UV Spectrum of Compound **1**.<br>UV Spectrum of compound 1.	11
15	**Figure S2** The (+)-HRMS(ESI) Spectroscopic Data of Compound **1**.	11
16	**Figure S3** The ^{1}H NMR Spectrum of Compound **1** in CD_3OD.	12
17	**Figure S4** The ^{13}C NMR Spectrum of Compound **1** in CD_3OD.	12
18	**Figure S5** The DEPT Spectrum of Compound **1** in CD_3OD.	13
19	**Figure S6** The HSQC Spectrum of Compound **1** in CD_3OD.	13
20	**Figure S7** The ^{1}H-^{1}H COSY Spectrum of Compound **1** in CD_3OD.	14
21	**Figure S8** The HMBC Spectrum of Compound **1** in CD_3OD.	14
22	**Figure S9** The NOESY Spectrum of Compound **1** in CD_3OD.	15
23	**Figure S10** The UV Spectrum of Compound **2**.	15
24	**Figure S11** The (+)-HRMS(ESI) Spectroscopic Data of Compound **2**.	16
25	**Figure S12** The ^{1}H NMR Spectrum of Compound **2** in DMSO-d_6.	16
26	**Figure S13** The ^{13}C NMR Spectrum of Compound **2** in DMSO-d_6.	17
27	**Figure S14** The DEPT Spectrum of Compound **2** in DMSO-d_6.	17
28	**Figure S15** The HSQC Spectrum of Compound **2** in DMSO-d_6.	18
29	**Figure S16** The ^{1}H-^{1}H COSY Spectrum of Compound **2** in DMSO-d_6.	18
30	**Figure S17** The HMBC Spectrum of Compound **2** in DMSO-d_6.	19
31	**Figure S18** The NOESY Spectrum of Compound **2** in DMSO-d_6.	19
32	**Figure S19** The UV Spectrum of Compound **3**.	20

33 **Figure S20** The (+)-HRMS(ESI) Spectroscopic Data of Compound **3**. **20**

34 **Figure S21** The ^{1}H NMR Spectrum of Compound **3** in CD_3OD. **21**

35 **Figure S22** The ^{13}C NMR Spectrum of Compound **3** in CD_3OD. **21**

36 **Figure S23** The DEPT Spectrum of Compound **3** in CD_3OD. **22**

37 **Figure S24** The HSQC Spectrum of Compound **3** in CD_3OD. **22**

38 **Figure S25** The ^{1}H-^{1}H COSY Spectrum of Compound **3** in CD_3OD. **23**

39 **Figure S26** The HMBC Spectrum of Compound **3** in CD_3OD. **23**

40 **Figure S27** The NOESY Spectrum of Compound **3** in CD_3OD. **24**

41 **Figure S28** The UV Spectrum of Compound **4**. **24**

42 **Figure S29** The (+)-HRMS(ESI) Spectroscopic Data of Compound **4**. **25**

43 **Figure S30** The ^{1}H NMR Spectrum of Compound **4** in CD_3OD. **25**

44 **Figure S31** The ^{13}C NMR Spectrum of Compound **4** in CD_3OD. **26**

45 **Figure S32** The DEPT Spectrum of Compound **4** in CD_3OD. **26**

46 **Figure S33** The HSQC Spectrum of Compound **4** in CD_3OD. **27**

47 **Figure S34** The ^{1}H-^{1}H COSY Spectrum of Compound **4** in CD_3OD. **27**

48 **Figure S35** The HMBC Spectrum of Compound **4** in CD_3OD. **28**

49 **Figure S36** The NOESY Spectrum of Compound **4** in CD_3OD. **28**

compound 1 autored

Table S1 Crystal data and structure refinement for compound **1** using autored.

Identification code	compound **1** autored
Empirical formula	$C_{24}H_{36}O_6$
Formula weight	420.53
Temperature/K	100.00(10)
Crystal system	monoclinic
Space group	$P2_1$
a/Å	9.33510(10)
b/Å	9.69210(10)
c/Å	13.5574(2)
α/°	90
β/°	107.5540(10)
γ/°	90
Volume/Å^3	1169.51(3)
Z	2
ρ_{calc}g/cm^3	1.194
μ/mm^{-1}	0.685
F(000)	456.0
Crystal size/mm^3	0.25 × 0.16 × 0.14
Radiation	Cu Kα (λ = 1.54184)
2Θ range for data collection/°	6.838 to 153.672
Index ranges	$-11 \le h \le 11$, $-11 \le k \le 12$, $-17 \le l \le 16$
Reflections collected	16082
Independent reflections	4731 [R_{int} = 0.0227, R_{sigma} = 0.0194]
Data/restraints/parameters	4731/1/279
Goodness-of-fit on F^2	1.042
Final R indexes [I>=2σ (I)]	R_1 = 0.0288, wR_2 = 0.0751
Final R indexes [all data]	R_1 = 0.0295, wR_2 = 0.0756
Largest diff. peak/hole / e Å^{-3}	0.28/-0.14
Flack parameter	0.00(5)

Table S2 Fractional atomic coordinates (×10[4]) and equivalent isotropic displacement parameters (Å[2]×10[3]) for compound **1** autored. U_{eq} is defined as 1/3 of of the trace of the orthogonalised U_{IJ} tensor.

**Atom**	***x***	***y***	***z***	**U(eq)**
O001	2457.5(13)	4042.4(13)	1040.6(9)	22.6(3)
O002	3094.1(14)	2236.3(13)	2975.8(10)	25.8(3)
O003	4507.5(15)	3551.4(14)	5157.2(10)	29.7(3)
O004	9359.3(17)	8640.3(15)	2526.8(12)	37.7(4)
O005	150.4(14)	4942.0(15)	765.2(12)	36.2(3)
O006	923.3(17)	1167.7(17)	2792.0(14)	44.8(4)
C007	5741.7(18)	4778.9(16)	2701.4(12)	18.1(3)
C008	5243.5(19)	5212.7(18)	774.1(13)	21.4(4)
C009	4270.0(18)	4887.2(16)	2597.7(12)	18.1(3)
C00A	4796(2)	4257.0(18)	4502.6(13)	22.1(4)
C00B	952.1(19)	4041(2)	624.4(14)	23.5(4)
C00C	7580.9(19)	6248.1(17)	2127.4(13)	20.0(3)
C00D	6455.8(18)	5023.1(17)	1835.8(12)	18.4(3)
C00E	6829.4(19)	4361.8(19)	3730.0(13)	22.3(4)
C00F	3890.3(19)	6028.2(19)	864.6(13)	22.3(3)
C00G	3156.9(19)	5303.3(17)	1575.9(13)	20.6(3)
C00H	8145.1(19)	8635.2(18)	2952.9(14)	23.8(4)
C00I	3600.4(19)	4611.9(17)	3484.7(14)	20.6(3)
C00J	2416(2)	3478.3(19)	3228.0(14)	23.4(4)
C00K	2193(2)	1139(2)	2748.6(14)	27.7(4)
C00L	6943.5(19)	7654.2(18)	2294.9(14)	22.8(4)
C00M	6319.9(19)	4852(2)	4640.0(13)	24.6(4)
C00N	5852(2)	5817(2)	-60.7(14)	28.8(4)
C00O	7378(2)	3725.7(18)	1758.4(14)	23.7(4)
C00P	2812(2)	5919(2)	3739.3(15)	27.0(4)
C00Q	8627(2)	8119(2)	4057.9(15)	27.7(4)
C00R	426(2)	2781(2)	-6.7(17)	34.4(5)
C00S	2946(2)	-88(2)	2456.0(15)	33.9(4)
C00T	9981(3)	7764(2)	4606.2(17)	39.6(5)
C00U	7496(2)	10083(2)	2949.1(18)	36.5(5)

Table S3 Anisotropic displacement parameters ($Å^2 \times 10^3$) for compound **1** autored. The anisotropic displacement factor exponent takes the form: $-2\pi^2[h^2a^{*2}U_{11}+2hka^*b^*U_{12}+\ldots]$.

Atom	U_{11}	U_{22}	U_{33}	U_{23}	U_{13}	U_{12}
O001	17.7(6)	22.5(6)	26.5(6)	-5.4(5)	5.0(5)	-2.1(5)
O002	25.7(6)	21.3(6)	32.6(7)	-1.5(5)	12.2(5)	-6.7(5)
O003	31.1(7)	33.7(8)	27.2(7)	7.4(6)	13.3(5)	-1.6(6)
O004	43.2(8)	33.4(8)	46.4(8)	-13.8(7)	28.5(7)	-21.3(7)
O005	21.0(6)	29.7(8)	54.9(9)	-0.8(7)	7.1(6)	1.5(6)
O006	34.5(8)	33.8(8)	69.5(12)	-6.8(8)	20.7(8)	-17.7(7)
C007	21.8(8)	13.0(7)	19.6(7)	-0.1(6)	6.5(6)	-3.6(6)
C008	22.9(8)	21.5(8)	19.7(8)	-1.9(6)	6.2(7)	-3.7(7)
C009	21.6(7)	13.0(7)	20.0(8)	-0.7(6)	7.1(6)	-3.1(6)
C00A	25.9(9)	20.6(8)	22.6(8)	-1.2(7)	11.7(7)	0.5(7)
C00B	18.2(8)	27.0(9)	25.4(8)	2.6(7)	6.9(6)	-2.6(7)
C00C	20.8(8)	19.0(8)	21.3(8)	-0.9(6)	8.0(6)	-4.3(6)
C00D	19.8(7)	16.8(8)	19.4(7)	0.0(6)	7.2(6)	-2.4(6)
C00E	20.4(8)	25.7(9)	21.7(8)	2.9(7)	7.6(7)	-1.0(7)
C00F	24.0(8)	21.4(8)	20.1(8)	2.3(6)	4.3(7)	-0.2(7)
C00G	20.5(8)	17.0(8)	23.9(8)	-1.9(6)	6.1(7)	-0.9(6)
C00H	24.0(8)	20.4(9)	27.4(9)	-3.4(7)	8.4(7)	-4.3(7)
C00I	20.0(8)	19.5(8)	23.6(8)	-1.0(6)	8.6(7)	-2.2(6)
C00J	22.3(8)	23.8(9)	27.0(8)	1.2(7)	11.8(7)	-3.1(7)
C00K	33.8(10)	25.3(9)	23.7(9)	3.0(7)	8.4(7)	-11.0(8)
C00L	23.3(8)	17.1(8)	26.4(8)	0.6(7)	5.1(7)	-1.9(7)
C00M	25.6(8)	27.5(9)	20.0(8)	-0.1(7)	6.0(7)	-3.2(8)
C00N	28.8(9)	38.2(11)	19.4(8)	2.0(8)	7.4(7)	-2.9(8)
C00O	25.5(8)	20.0(8)	27.0(9)	-2.4(7)	10.1(7)	-1.5(7)
C00P	28.8(9)	26.5(9)	28.0(9)	-1.0(7)	12.3(7)	3.2(8)
C00Q	28.6(9)	27.2(10)	28.0(9)	-5.4(7)	9.7(8)	-1.1(7)
C00R	23.2(9)	41.0(12)	37.7(11)	-12.0(9)	7.1(8)	-8.7(8)
C00S	47.3(11)	26.2(10)	30.6(10)	-1.6(8)	15.3(9)	-8.3(9)
C00T	40.7(11)	36.9(11)	34.9(11)	-6.3(9)	1.7(9)	4.9(9)
C00U	36.4(10)	19.6(9)	45.5(12)	-3.8(9)	0.4(9)	-2.0(8)

Table S4 Bond Lengths for compound **1** determined using autored.

**Atom**	**Atom**	**Length/Å**	**Atom**	**Atom**	**Length/Å**
O001	C00B	1.347(2)	C00A	C00I	1.530(2)
O001	C00G	1.470(2)	C00A	C00M	1.493(2)
O002	C00J	1.448(2)	C00B	C00R	1.488(3)
O002	C00K	1.333(2)	C00C	C00D	1.555(2)
O003	C00A	1.213(2)	C00C	C00L	1.531(2)
O004	C00H	1.419(2)	C00D	C00O	1.545(2)
O005	C00B	1.202(2)	C00E	C00M	1.526(2)
O006	C00K	1.204(2)	C00F	C00G	1.514(2)
C007	C009	1.342(2)	C00H	C00L	1.534(2)
C007	C00D	1.533(2)	C00H	C00Q	1.514(3)
C007	C00E	1.512(2)	C00H	C00U	1.528(3)
C008	C00D	1.550(2)	C00I	C00J	1.523(2)
C008	C00F	1.526(2)	C00I	C00P	1.555(2)
C008	C00N	1.528(2)	C00K	C00S	1.495(3)
C009	C00G	1.514(2)	C00Q	C00T	1.304(3)
C009	C00I	1.537(2)			

Table S5 Bond Angles for compound **1** determined using autored.

Atom	Atom	Atom	Angle/°	Atom	Atom	Atom	Angle/°
C00B	O001	C00G	117.34(13)	C007	C00E	C00M	112.10(14)
C00K	O002	C00J	115.27(14)	C00G	C00F	C008	110.81(14)
C009	C007	C00D	125.14(15)	O001	C00G	C009	108.08(13)
C009	C007	C00E	119.93(14)	O001	C00G	C00F	106.87(14)
C00E	C007	C00D	114.92(14)	C00F	C00G	C009	112.89(14)
C00F	C008	C00D	112.14(13)	O004	C00H	C00L	107.40(14)
C00F	C008	C00N	111.26(15)	O004	C00H	C00Q	111.62(15)
C00N	C008	C00D	113.76(14)	O004	C00H	C00U	111.10(16)
C007	C009	C00G	120.99(14)	C00Q	C00H	C00L	108.52(15)
C007	C009	C00I	123.34(14)	C00Q	C00H	C00U	107.85(16)
C00G	C009	C00I	115.67(13)	C00U	C00H	C00L	110.34(15)
O003	C00A	C00I	121.59(16)	C009	C00I	C00P	111.33(14)
O003	C00A	C00M	122.68(17)	C00A	C00I	C009	112.74(13)
C00M	C00A	C00I	115.73(14)	C00A	C00I	C00P	104.38(14)
O001	C00B	C00R	111.31(15)	C00J	C00I	C009	112.66(14)
O005	C00B	O001	123.63(17)	C00J	C00I	C00A	109.03(14)
O005	C00B	C00R	125.05(16)	C00J	C00I	C00P	106.18(14)
C00L	C00C	C00D	117.02(14)	O002	C00J	C00I	108.33(13)
C007	C00D	C008	111.40(13)	O002	C00K	C00S	112.42(16)
C007	C00D	C00C	109.85(13)	O006	C00K	O002	122.18(19)
C007	C00D	C00O	107.83(13)	O006	C00K	C00S	125.40(18)
C008	C00D	C00C	113.17(13)	C00C	C00L	C00H	112.90(14)
C00O	C00D	C008	107.40(13)	C00A	C00M	C00E	107.22(14)
C00O	C00D	C00C	106.93(13)	C00T	C00Q	C00H	126.8(2)

Table S6 Hydrogen Bonds for compound **1** determined using autored.

D	H	A	d(D-H)/Å	d(H-A)/Å	d(D-A)/Å	D-H-A/°
O004	H004	O006[1]	0.84	1.98	2.819(2)	173.9

[1]1+X,1+Y,+Z

Table S7 Torsion Angles for compound **1** determined using autored.

A	B	C	D	Angle/°	A	B	C	D	Angle/°
O003	C00A	C00I	C009	-153.05(16)	C00E	C007	C00D	C00O	53.22(18)
O003	C00A	C00I	C00J	-27.1(2)	C00F	C008	C00D	C007	37.87(19)
O003	C00A	C00I	C00P	86.0(2)	C00F	C008	C00D	C00C	-86.48(17)
O003	C00A	C00M	C00E	123.88(18)	C00F	C008	C00D	C00O	155.74(14)
O004	C00H	C00L	C00C	49.8(2)	C00G	O001	C00B	O005	-8.6(2)
O004	C00H	C00Q	C00T	2.8(3)	C00G	O001	C00B	C00R	172.49(15)
C007	C009	C00G	O001	98.11(17)	C00G	C009	C00I	C00A	-177.57(14)
C007	C009	C00G	C00F	-19.9(2)	C00G	C009	C00I	C00J	58.50(19)
C007	C009	C00I	C00A	2.2(2)	C00G	C009	C00I	C00P	-60.65(18)
C007	C009	C00I	C00J	-121.74(17)	C00I	C009	C00G	O001	-82.13(17)
C007	C009	C00I	C00P	119.10(17)	C00I	C009	C00G	C00F	159.88(14)
C007	C00E	C00M	C00A	57.73(19)	C00I	C00A	C00M	C00E	-57.1(2)
C008	C00F	C00G	O001	-69.16(17)	C00J	O002	C00K	O006	-2.9(3)
C008	C00F	C00G	C009	49.54(19)	C00J	O002	C00K	C00S	177.75(15)
C009	C007	C00D	C008	-8.1(2)	C00K	O002	C00J	C00I	178.96(14)
C009	C007	C00D	C00C	118.15(17)	C00L	C00C	C00D	C007	-60.81(19)
C009	C007	C00D	C00O	-125.66(17)	C00L	C00C	C00D	C008	64.39(19)
C009	C007	C00E	C00M	-31.3(2)	C00L	C00C	C00D	C00O	-177.57(14)
C009	C00I	C00J	O002	57.43(19)	C00L	C00H	C00Q	C00T	120.9(2)
C00A	C00I	C00J	O002	-68.52(17)	C00M	C00A	C00I	C009	27.9(2)
C00B	O001	C00G	C009	133.55(14)	C00M	C00A	C00I	C00J	153.83(15)
C00B	O001	C00G	C00F	-104.67(16)	C00M	C00A	C00I	C00P	-93.05(17)
C00D	C007	C009	C00G	-1.2(2)	C00N	C008	C00D	C007	165.23(15)
C00D	C007	C009	C00I	179.08(15)	C00N	C008	C00D	C00C	40.9(2)
C00D	C007	C00E	C00M	149.77(15)	C00N	C008	C00D	C00O	-76.90(18)
C00D	C008	C00F	C00G	-59.71(19)	C00N	C008	C00F	C00G	171.60(14)
C00D	C00C	C00L	C00H	158.46(14)	C00P	C00I	C00J	O002	179.53(13)
C00E	C007	C009	C00G	180.00(15)	C00Q	C00H	C00L	C00C	-71.02(19)
C00E	C007	C009	C00I	0.3(2)	C00U	C00H	C00L	C00C	171.02(16)
C00E	C007	C00D	C008	170.82(14)	C00U	C00H	C00Q	C00T	-119.5(2)
C00E	C007	C00D	C00C	-62.97(18)					

Table S8 Hydrogen Atom Coordinates (Å×104) and Isotropic Displacement Parameters (Å2×10^{3}) for compound **1** determined using autored.

**Atom**	***x***	***y***	***z***	**U(eq)**
H004	9766.83	9422.05	2610.15	57
H008	4865.79	4268.03	532.75	26
H00A	8379.92	5999.6	2769.27	24
H00B	8062.73	6349.87	1573.37	24
H00C	7830.86	4755.98	3791.23	27
H00D	6926.84	3344.3	3756.48	27
H00E	4217.87	6961.15	1137.94	27
H00F	3150.99	6133.11	170.66	27
H00G	2367.3	5912.44	1704.69	25
H00H	2044.02	3308.51	3828.32	28
H00I	1554.33	3760.08	2633.32	28
H00J	6157.56	7515.83	2638.91	27
H00K	6463.31	8087.02	1613.7	27
H00L	7031.8	4533.74	5300.61	29
H00M	6276.84	5872.62	4650.21	29
H00N	6106.78	6790.67	92.2	43
H00O	5085.58	5739.97	-736.07	43
H00P	6752.95	5308.13	-73.8	43
H00Q	8187.13	3601.04	2408.48	35
H00R	7810.94	3838.62	1188.63	35
H00S	6720.55	2914.61	1628.97	35
H00T	3517.29	6695.34	3879.35	40
H00U	2485.1	5741.33	4349.86	40
H00V	1937.19	6143.41	3149.17	40
H00W	7864.3	8045.42	4386.59	33
H00X	561.94	2898.96	-690.67	52
H00Y	-640.85	2629.47	-84.46	52
H	1008.93	1982.79	339.32	52
H00Z	3381.58	169.93	1908.89	51
HA	2206.13	-824.85	2205.26	51
HB	3743.15	-414.49	3062.16	51
H00	10785.65	7817.43	4314.47	48
HC	10162.43	7451.25	5297.3	48
H1	8276.5	10699.6	3367.81	55
HD	6654.43	10049.69	3239.6	55
HE	7141.48	10428.91	2237.28	55

Computational Section

Spartan 14 program (Wavefunction Inc., Irvine, CA, USA) was used for calculating Merck molecular force field (MMFF). Gaussian 16 program package1 was used for density functional theory (DFT) and time-dependent density functional theory (TDDFT) calculations. The conformational search was performed by a MMFF model, then the conformers with lower relative energies (< 10 kcal/mol) were subjected to geometry optimization with the DFT method at the B3LYP/6-31G* level. Vibrational frequency calculations were done at the same level to evaluate their relative thermal (ΔE) and free energies (ΔG) at 298.15 K. To obtain the energies of these lower energy conformers, the geometry optimized conformers were further calculated at the B3LYP/6-311G* level was taken into consideration using SMD. The specific optical rotation calculations for compounds were performed using the foregoing lower energy conformers at the B3LYP/6-311G* level, all of which were subjected to specific optical rotation calculations at the B3LYP/6-311+G(d) level in MeOH with SMD model. The calculated specific optical rotation data of these conformers were averaged according to the Boltzmann distribution theory and their relative Gibbs free energy.

Table S9. Energies of the dominative conformers of compound **1**.

Compound	No.	Structure	*E* (Hartree)	Population (%)
**1**	C1	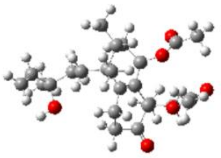	-1387.74167452	80.97
	C2	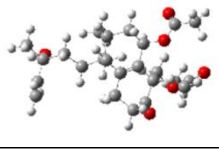	-1387.73608350	0.15
	C3	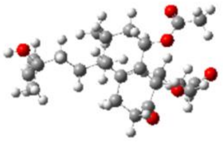	-1387.73314361	0.01

	C4	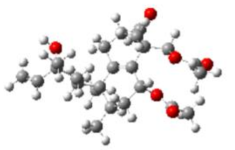	-1387.74141520	6.97
	C5	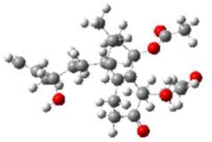	-1387.73991870	11.91

Table S10. Energies of the dominative conformers of compounds **2** and **3**.

compounds	No.	structure	*E*_6-311g(d)	*G*
**2**	C1	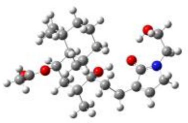	-1369.2434281	-1368.7022451
	C2	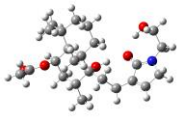	-1369.2434281	-1368.7022451
	C3	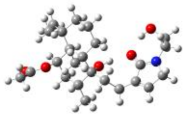	-1369.2432885	-1368.7021805
	C4	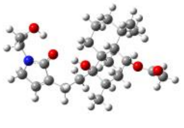	-1369.2432885	-1368.7021665
	C5	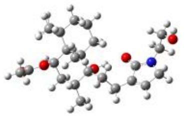	-1369.2417468	-1368.7018458

	C6	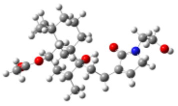	-1369.2418376	-1368.7017786
**3**	C1	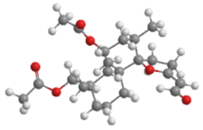	-1348.6309858	-1348.1570218
	C2	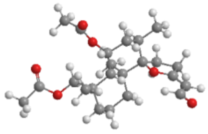	-1348.6309851	-1348.1570031
	C3	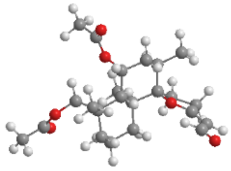	-1348.6307963	-1348.1564193
	C4	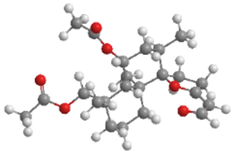	-1348.6313701	-1348.1573801

Table S11. Calculated and measured OR values of compounds **2, 3** and **4** at different wavelengths.

compounds	Rotation value	$[\alpha]_{633}$	$[\alpha]_{589}$	$[\alpha]_{546}$	$[\alpha]_{436}$	$[\alpha]_{365}$
**2**	$[\alpha]_D$ calcd.	-81.50	-96.42	-115.63	-206.95	-355.04
	$[\alpha]_D$ exptl.	34.48	51.77	64.37	187.27	234.83
**3**	$[\alpha]_D$ calcd.	-34.65	-41.33	-49.97	-89.34	-92.72
	$[\alpha]_D$ exptl.	19.04	24.84	27.78	49.82	69.03
**4**	$[\alpha]_D$ exptl.	7.08	20.53	24.52	29.60	37.20

Table S12 The docking pockets

Receptor	Pocket Position	Pocket size
INOS	center_x = 122.834	size_x = 30
	center_y = 114.553	size_y = 30
	center_z = 36.778	size_z = 30
COX-2	center_x = 26.999	size_x = 30
	center_y = 23.927	size_y = 30
	center_z = 15.269	size_z = 30

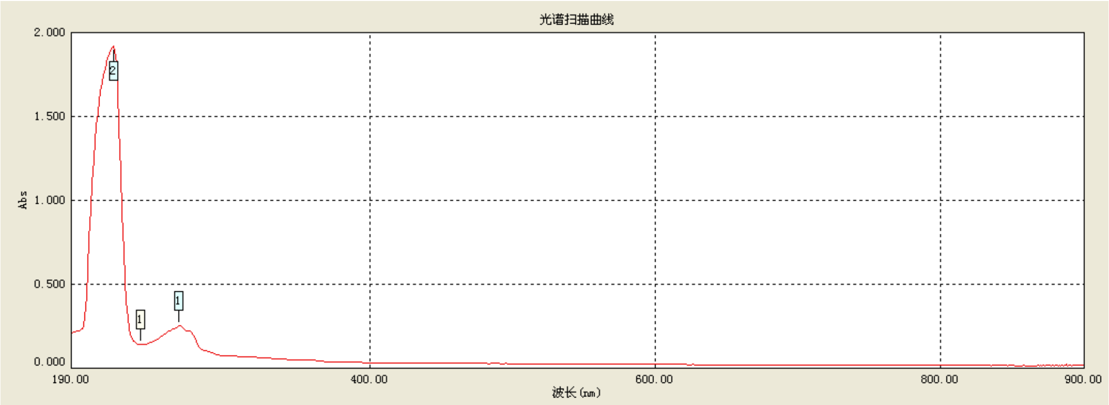

Figure S1 The UV Spectrum of Compound **1**.

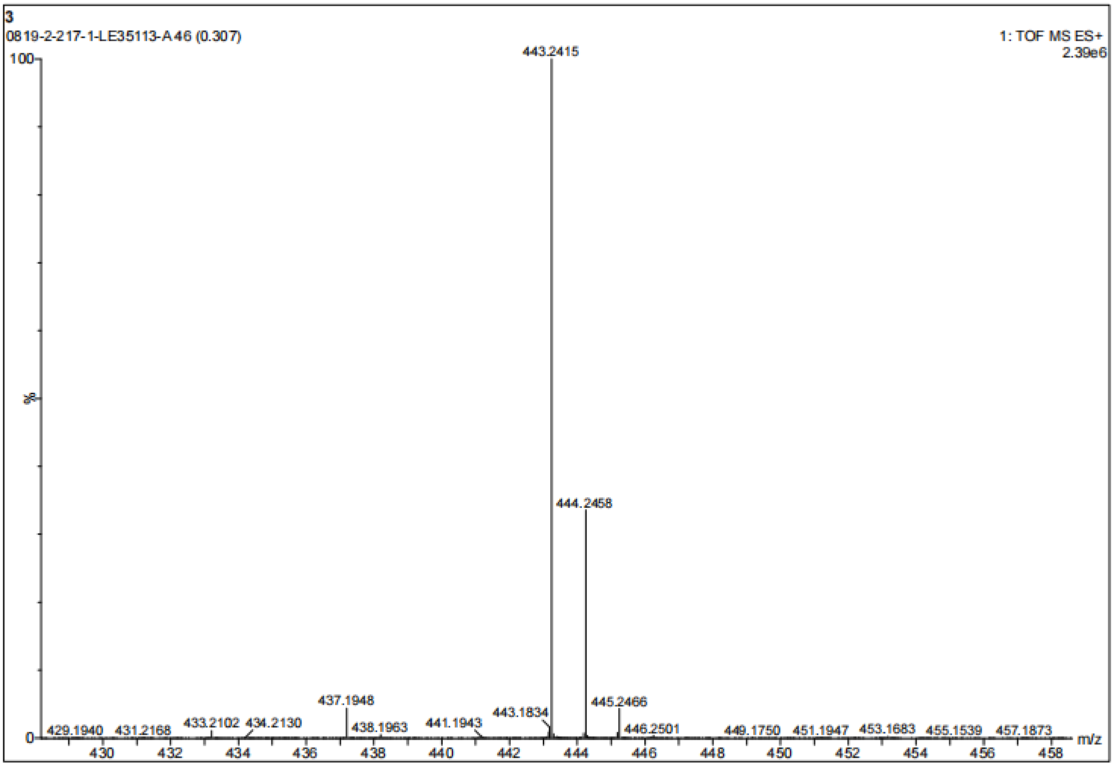

Figure S2 The (+)-HRMS(ESI) Spectroscopic Data of Compound **1**.

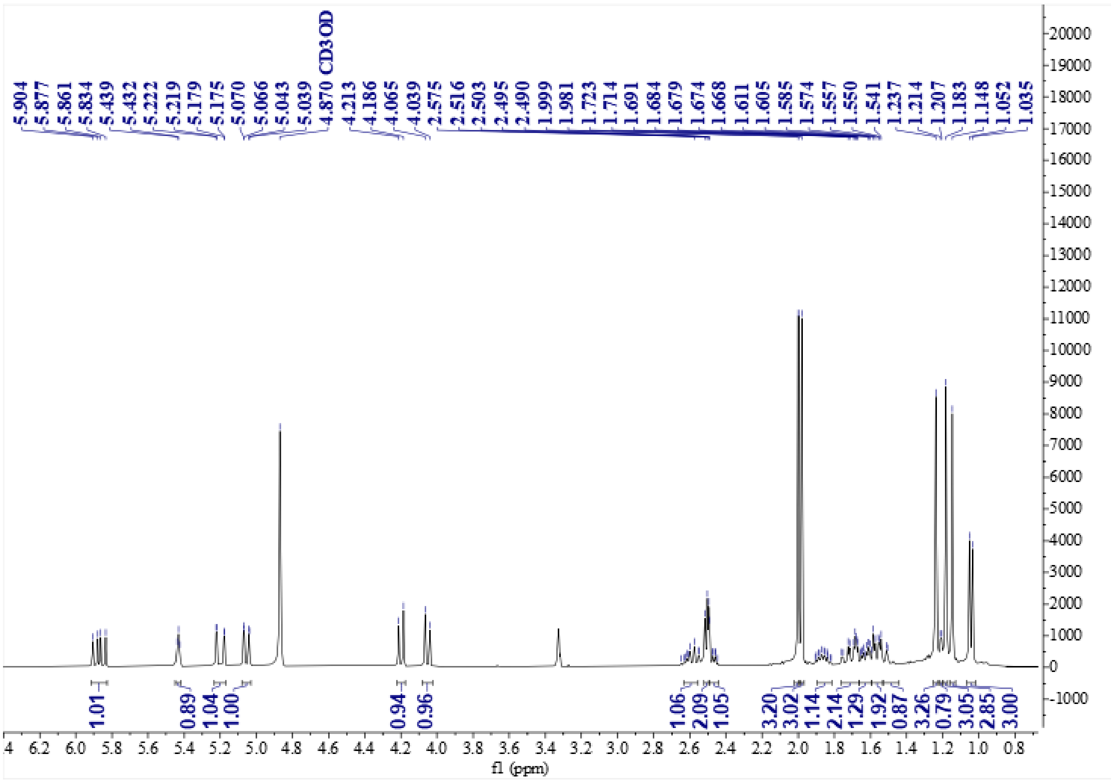

Figure S3 The ^{1}H NMR Spectrum of Compound **1** in CD_3OD.

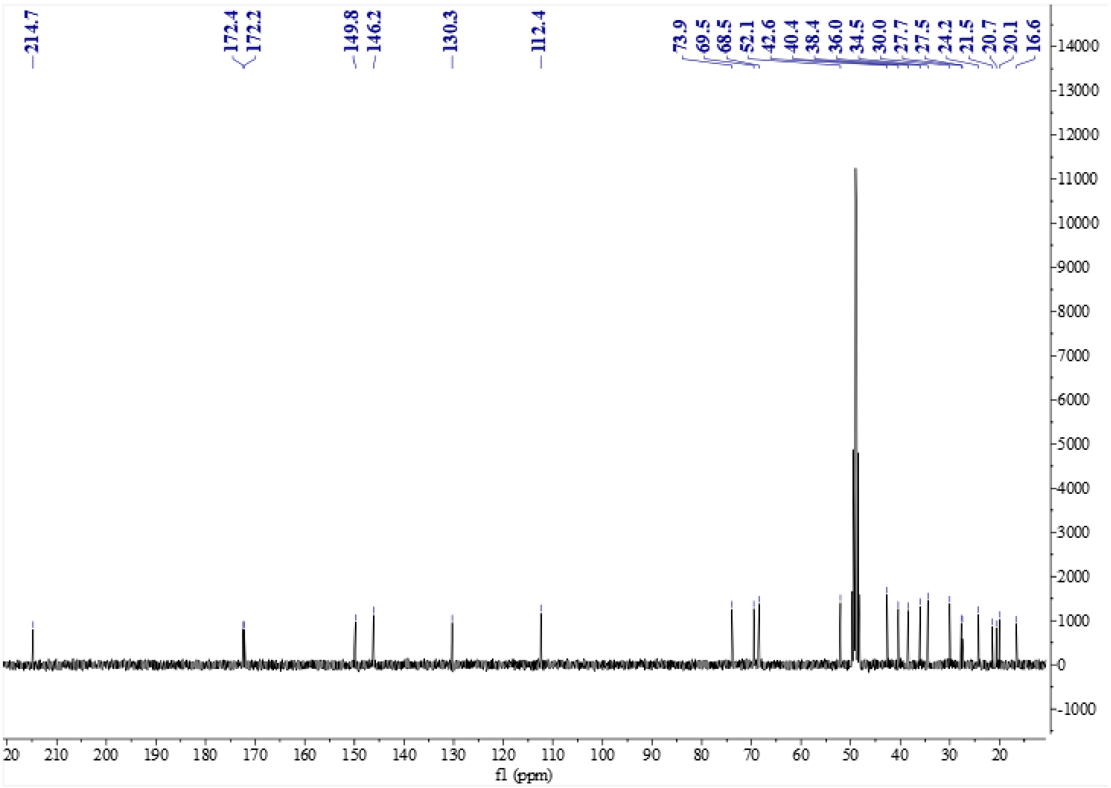

Figure S4 The ^{13}C NMR Spectrum of Compound **1** in CD_3OD.

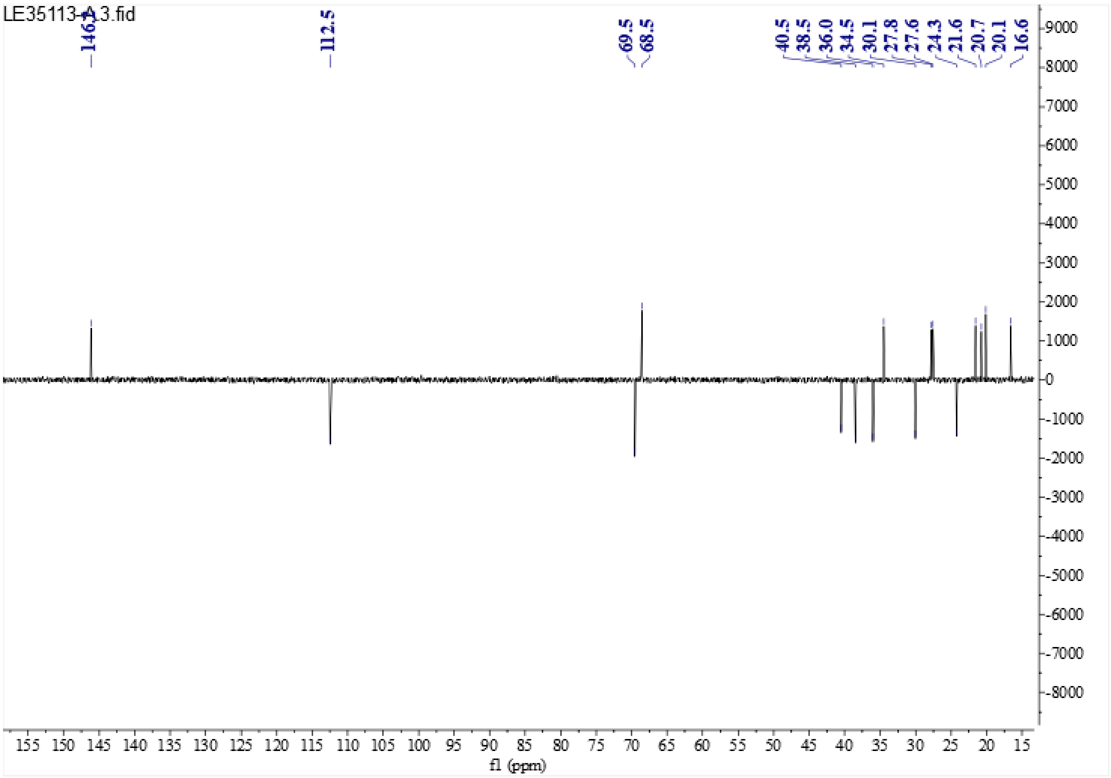

Figure S5 The DEPT Spectrum of Compound **1** in CD_3OD.

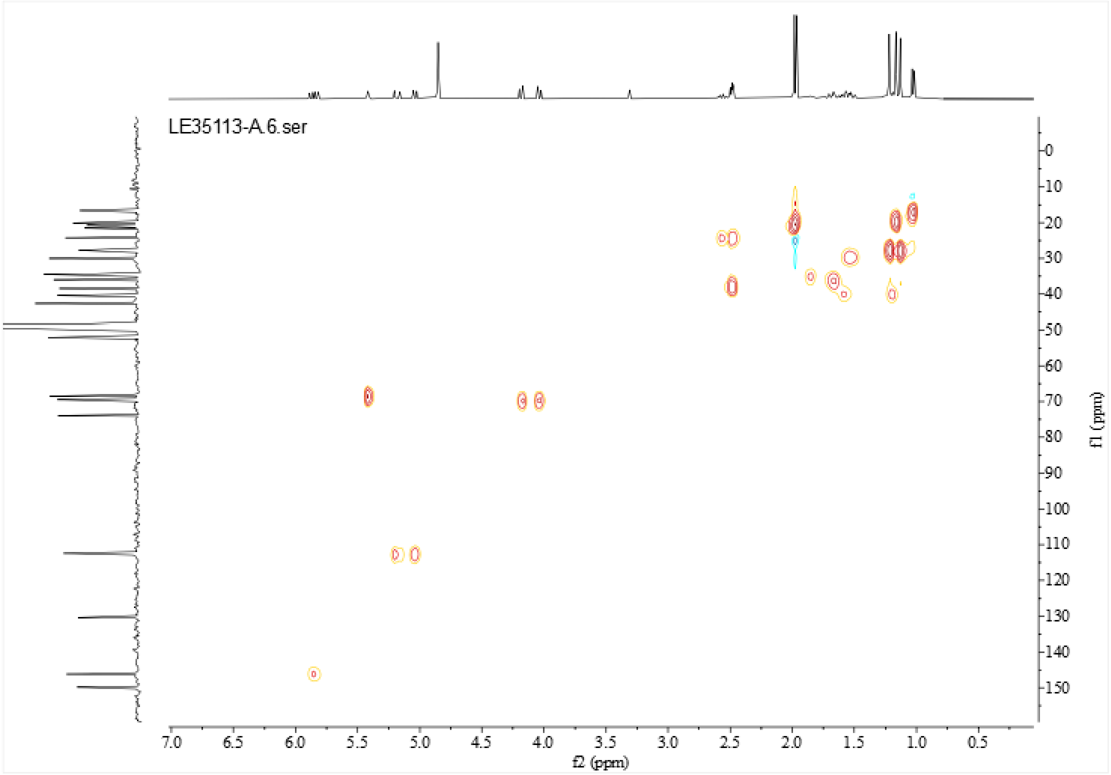

Figure S6 The HSQC Spectrum of Compound **1** in CD_3OD.

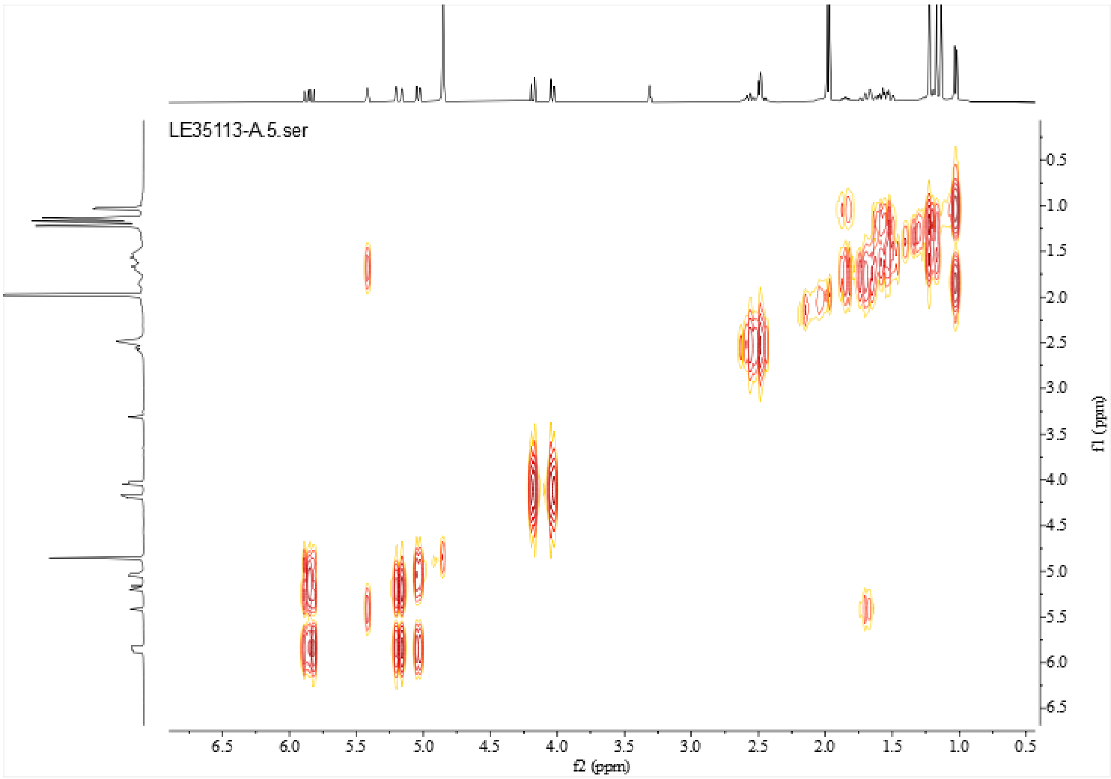

Figure S7 The ^{1}H-^{1}H COSY Spectrum of Compound **1** in CD_3OD.

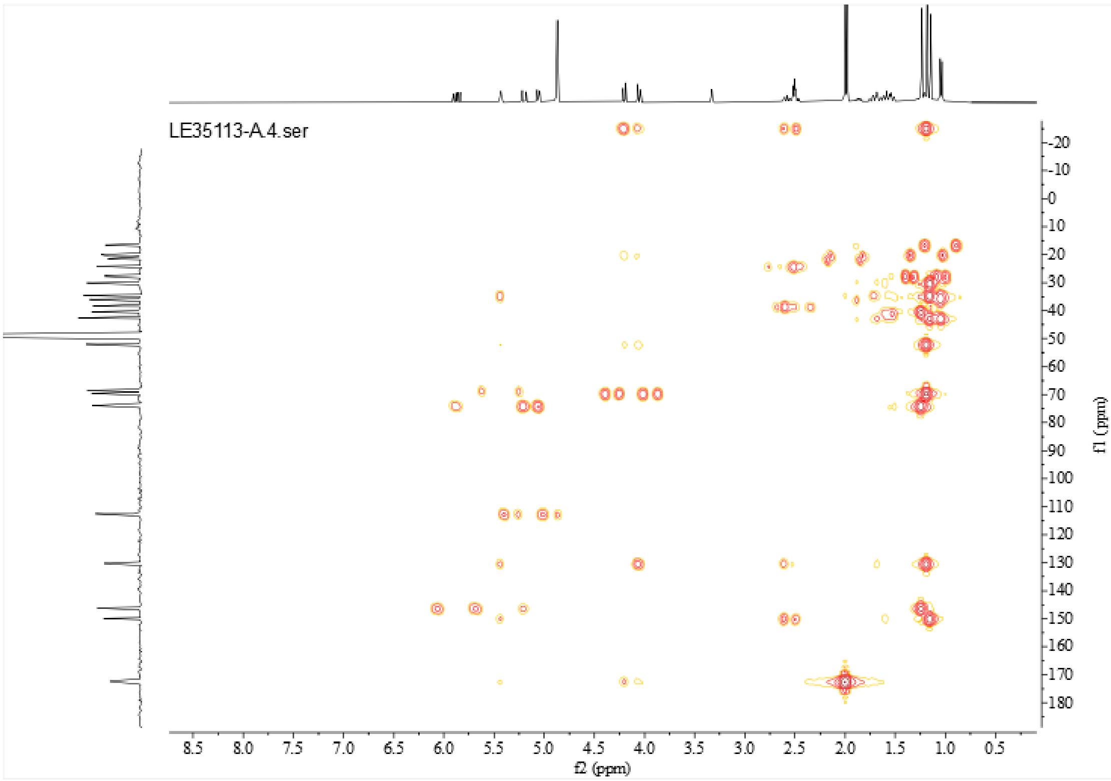

Figure S8 The HMBC Spectrum of Compound **1** in CD_3OD.

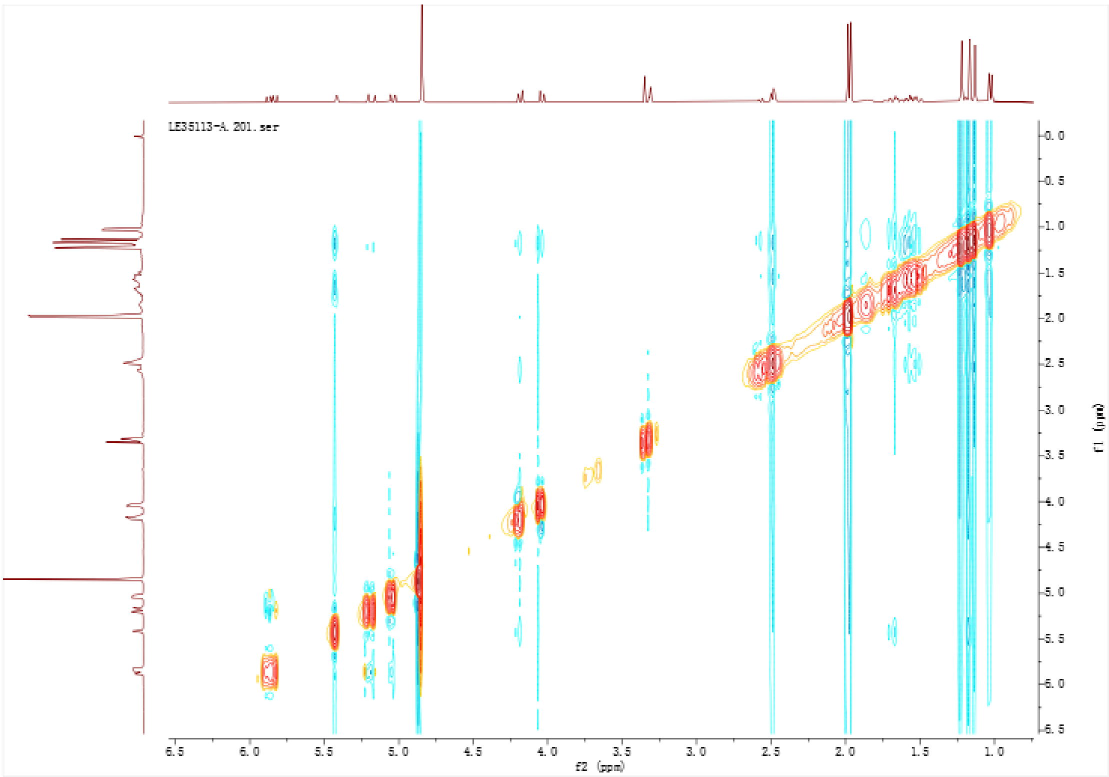

Figure S9 The NOESY Spectrum of Compound **1** in CD_3OD.

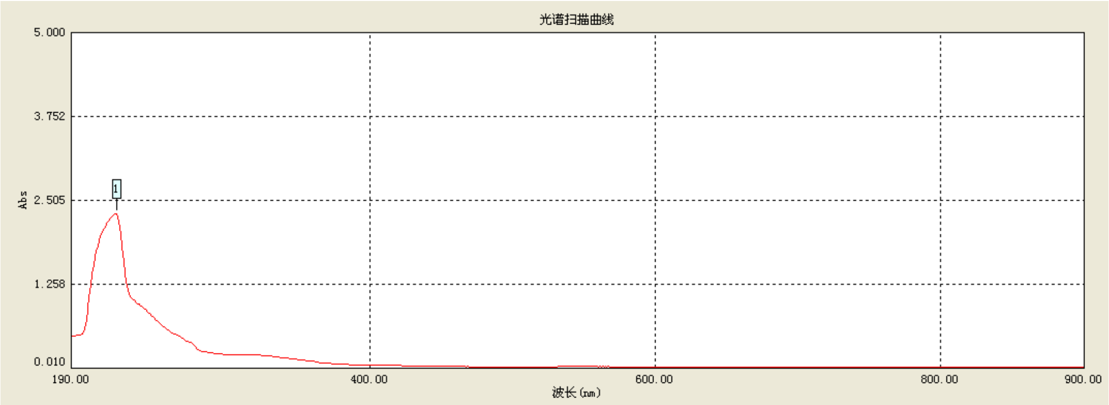

Figure S10 The UV Spectrum of Compound **2**.

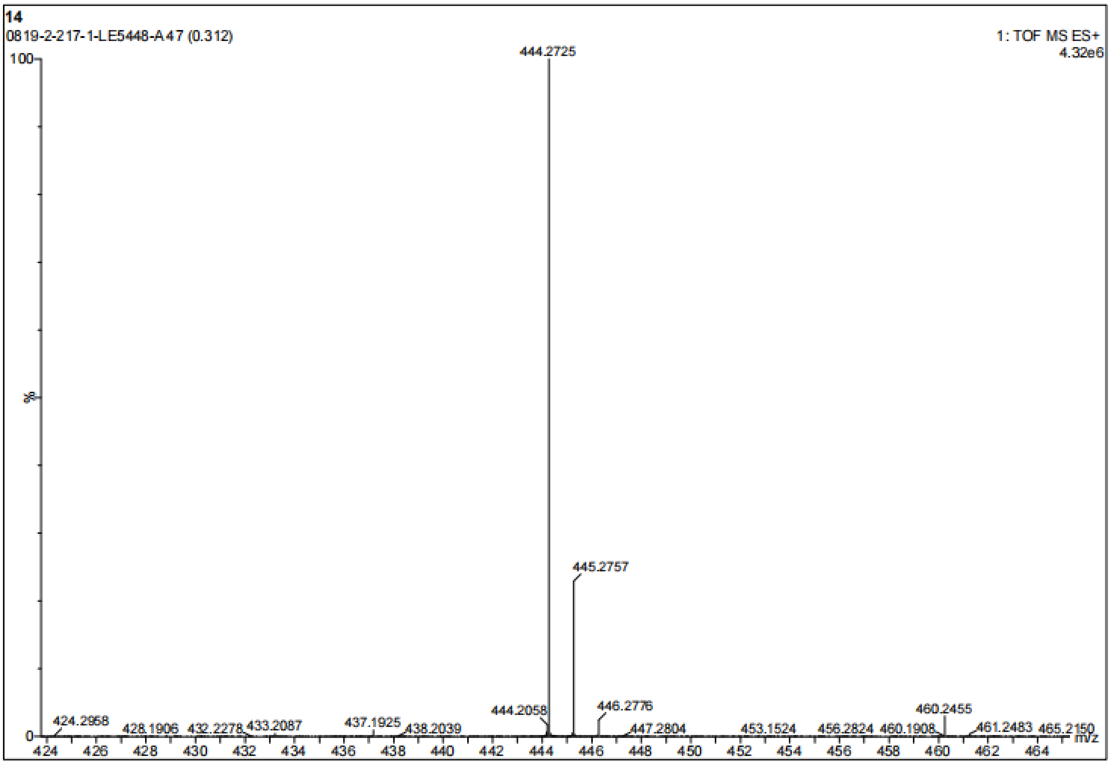

Figure S11 The (+)-HRMS(ESI) Spectroscopic Data of Compound **2**.

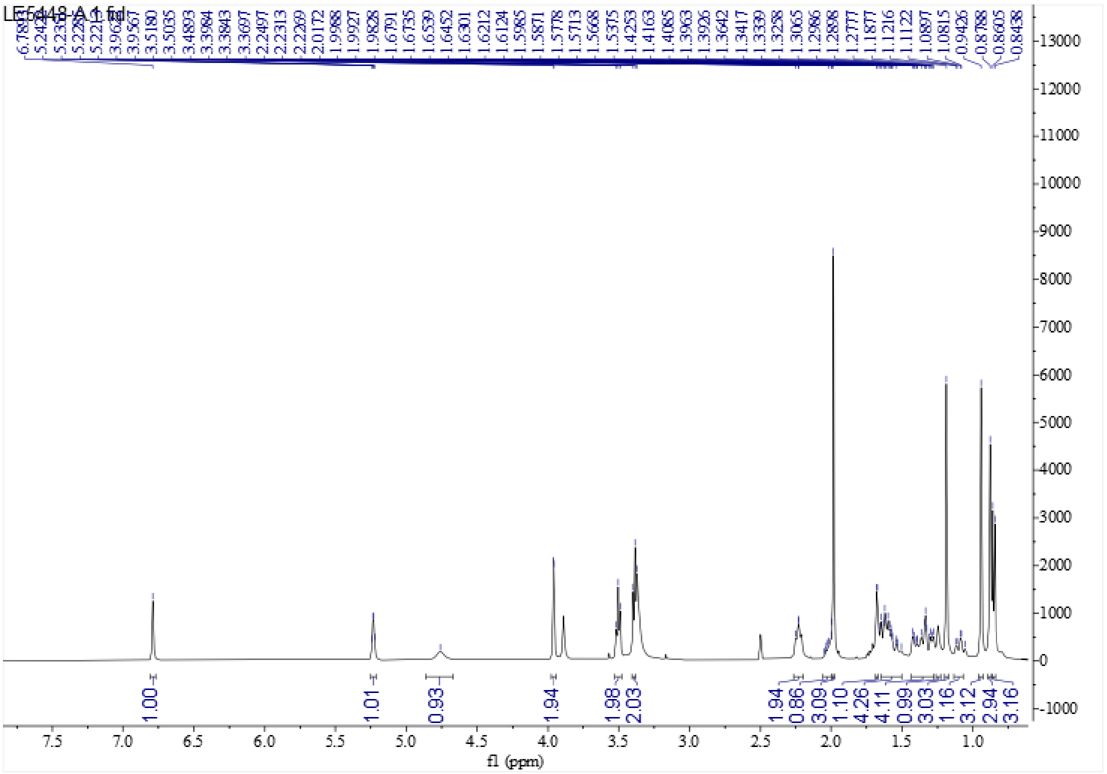

Figure S12 The ^{1}H NMR Spectrum of Compound **2** in DMSO-d_6.

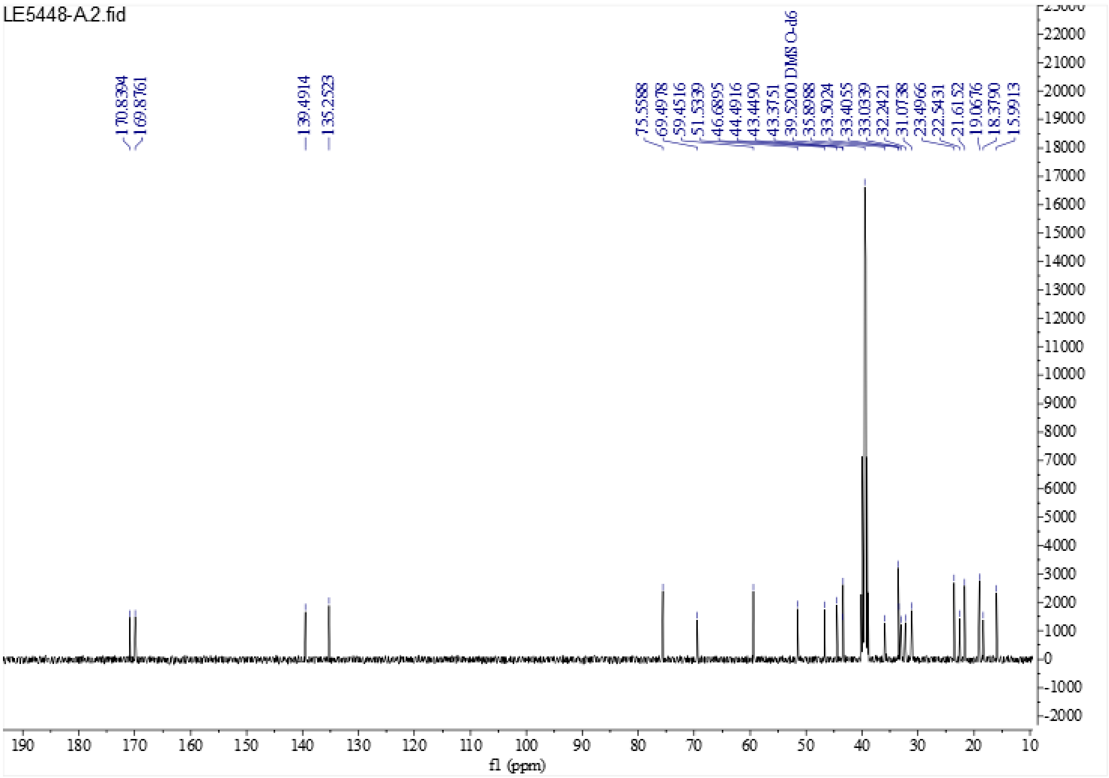

Figure S13 The ^{13}C NMR Spectrum of Compound **2** in DMSO-d_6.

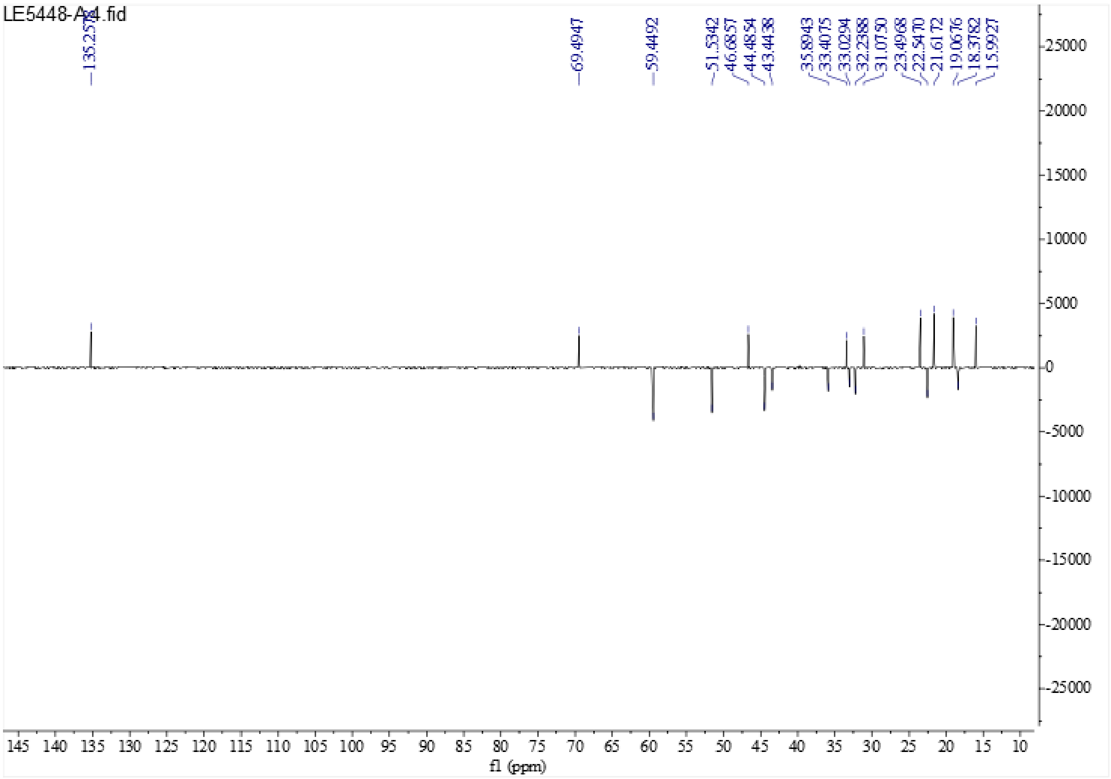

Figure S14 The DEPT Spectrum of Compound **2** in DMSO-d_6.

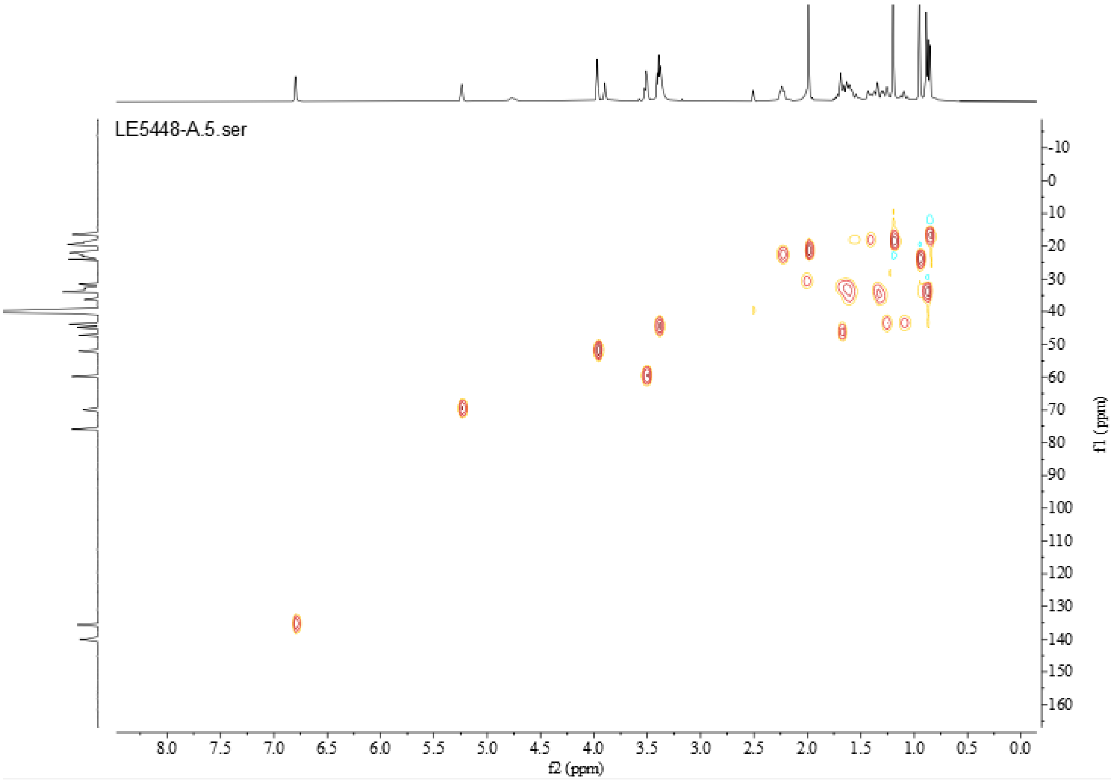

Figure S15 The HSQC Spectrum of Compound **2** in DMSO-d_6.

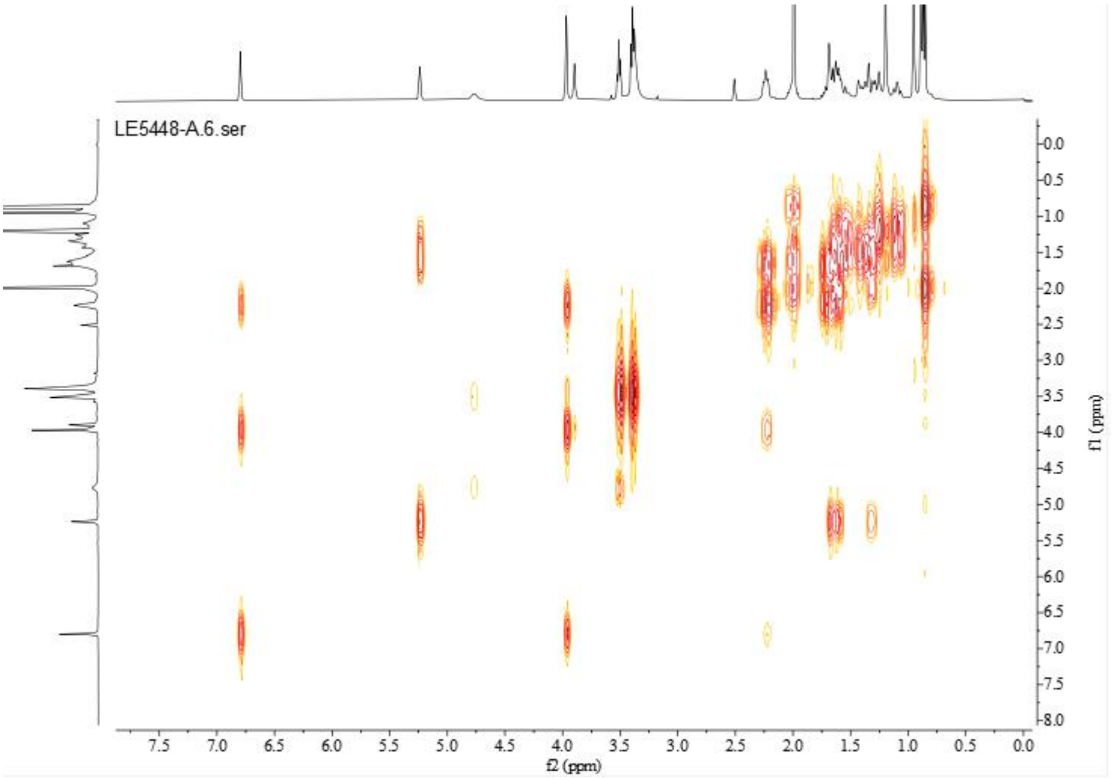

Figure S16 The ^{1}H-^{1}H COSY Spectrum of Compound **2** in DMSO-d_6.

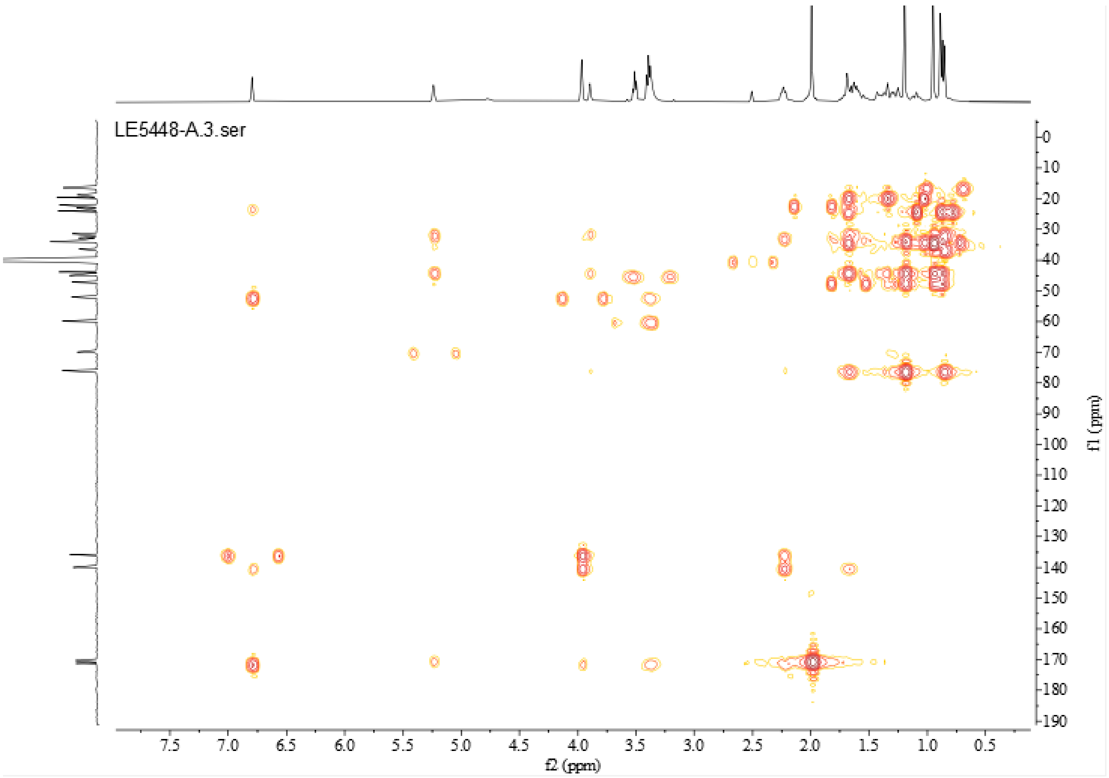

Figure S17 The HMBC Spectrum of Compound **2** in DMSO-d_6.

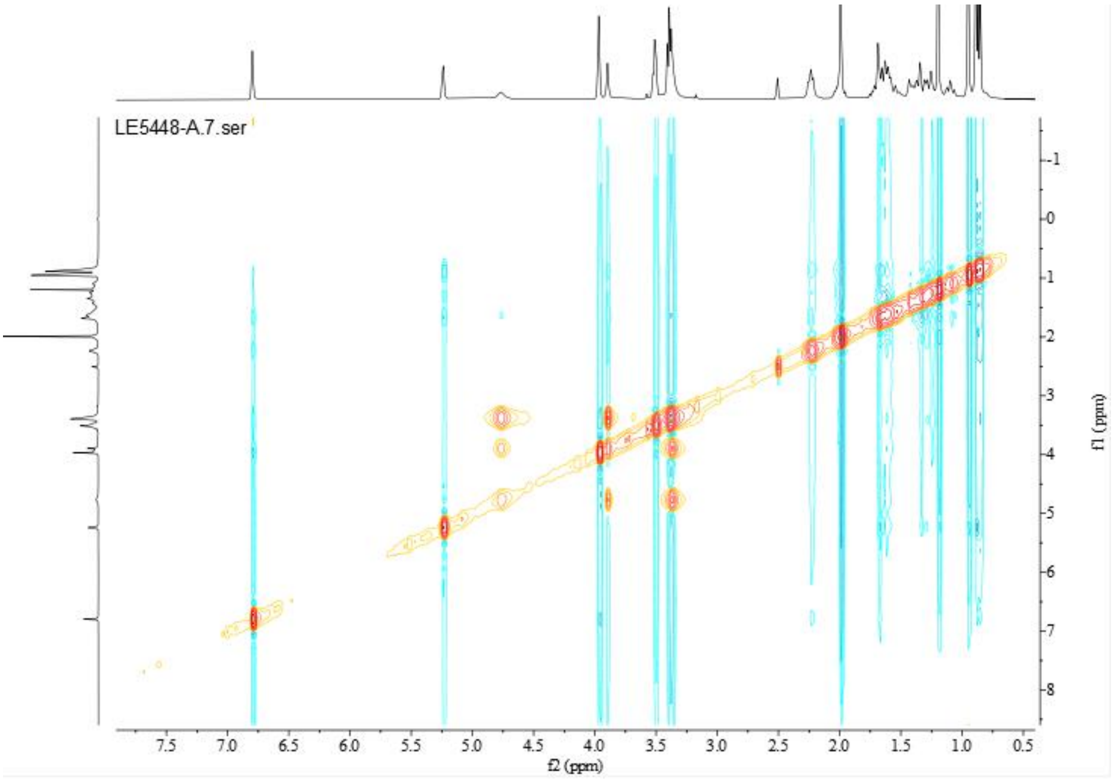

Figure S18 The NOESY Spectrum of Compound **2** in DMSO-d_6.

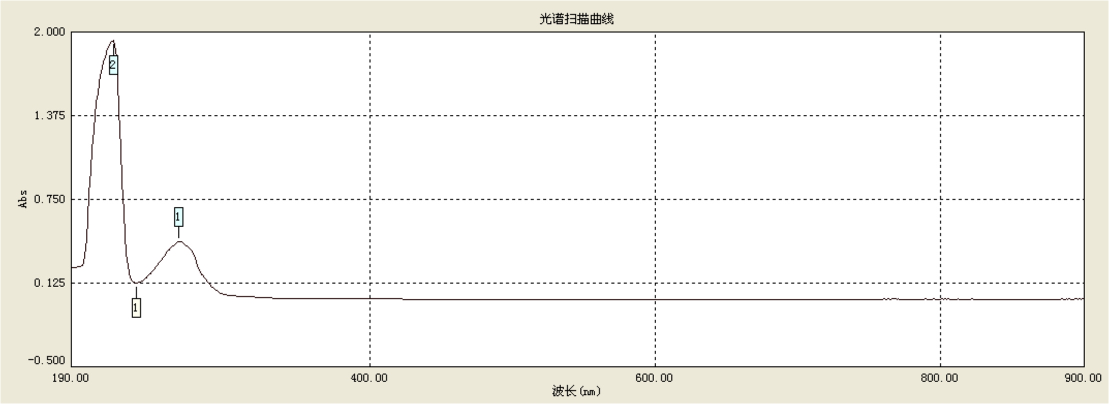

Figure S19 The UV Spectrum of Compound **3**.

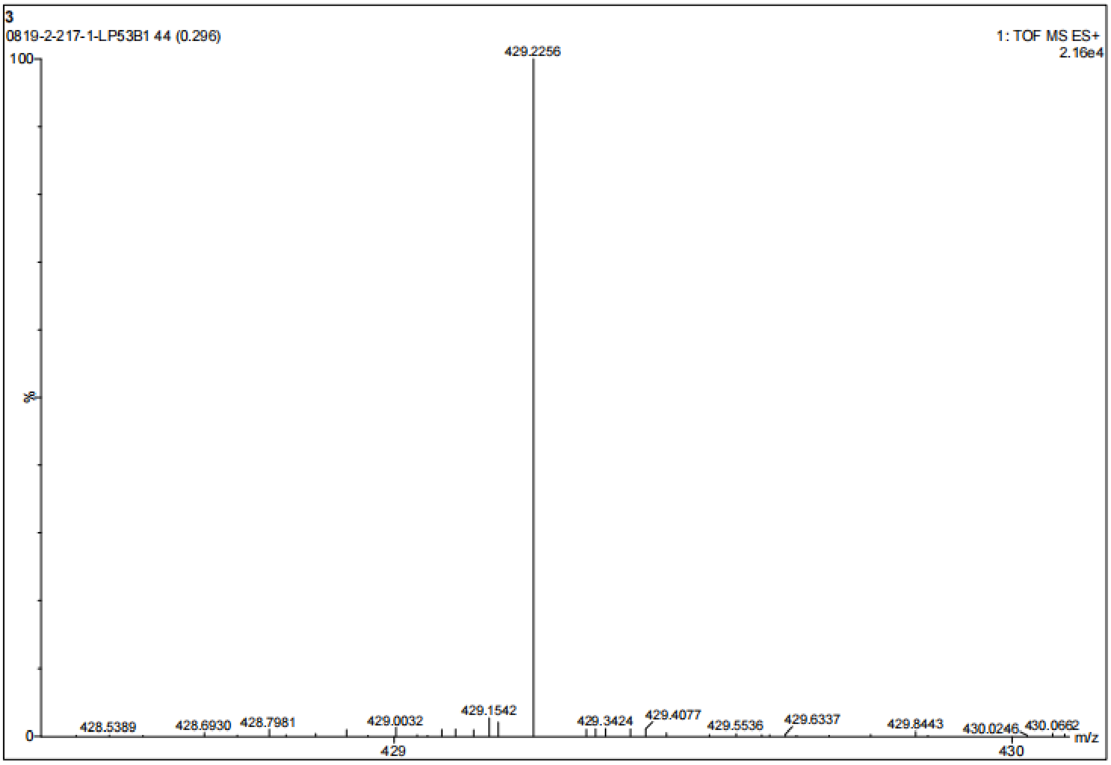

Figure S20 The (+)-HRMS(ESI) Spectroscopic Data of Compound **3**.

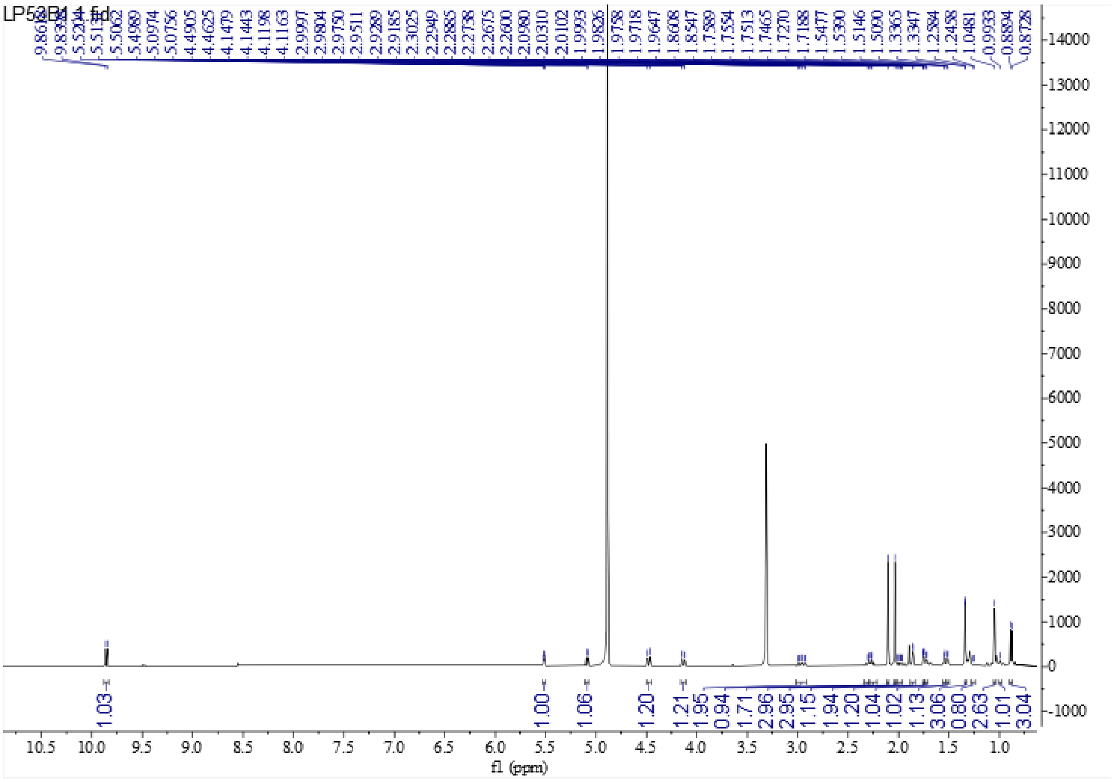

Figure S21 The ^{1}H NMR Spectrum of Compound **3** in CD_3OD.

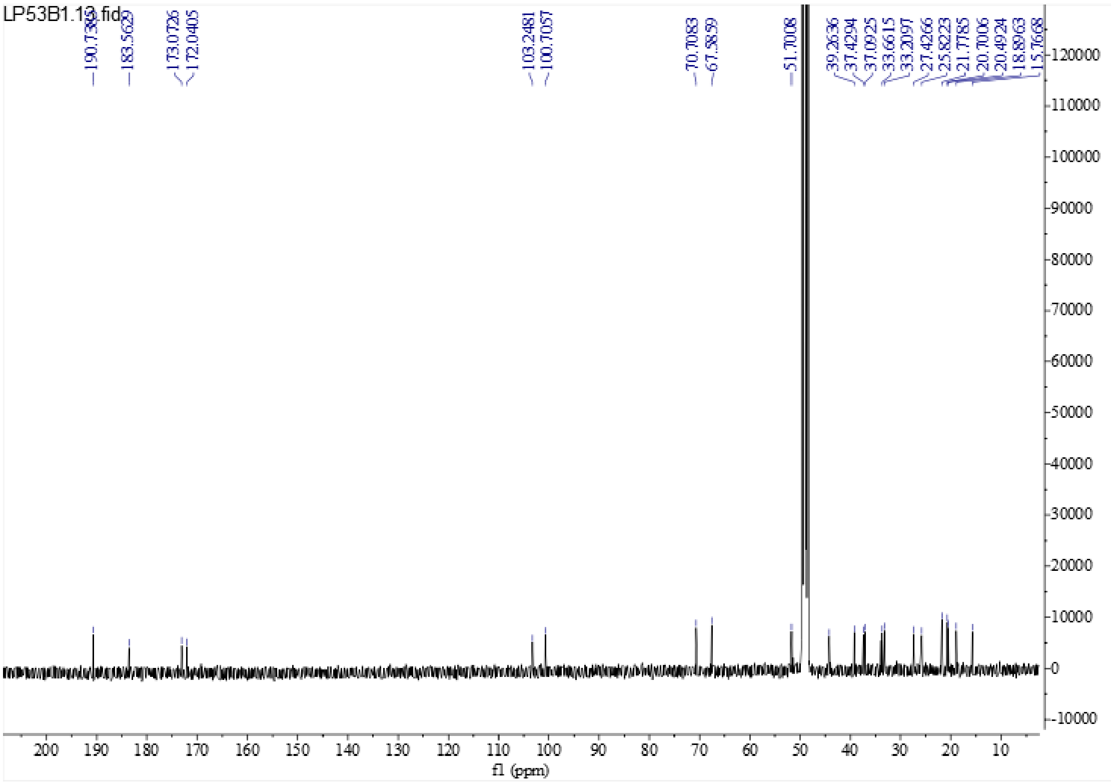

Figure S22 The ^{13}C NMR Spectrum of Compound **3** in CD_3OD.

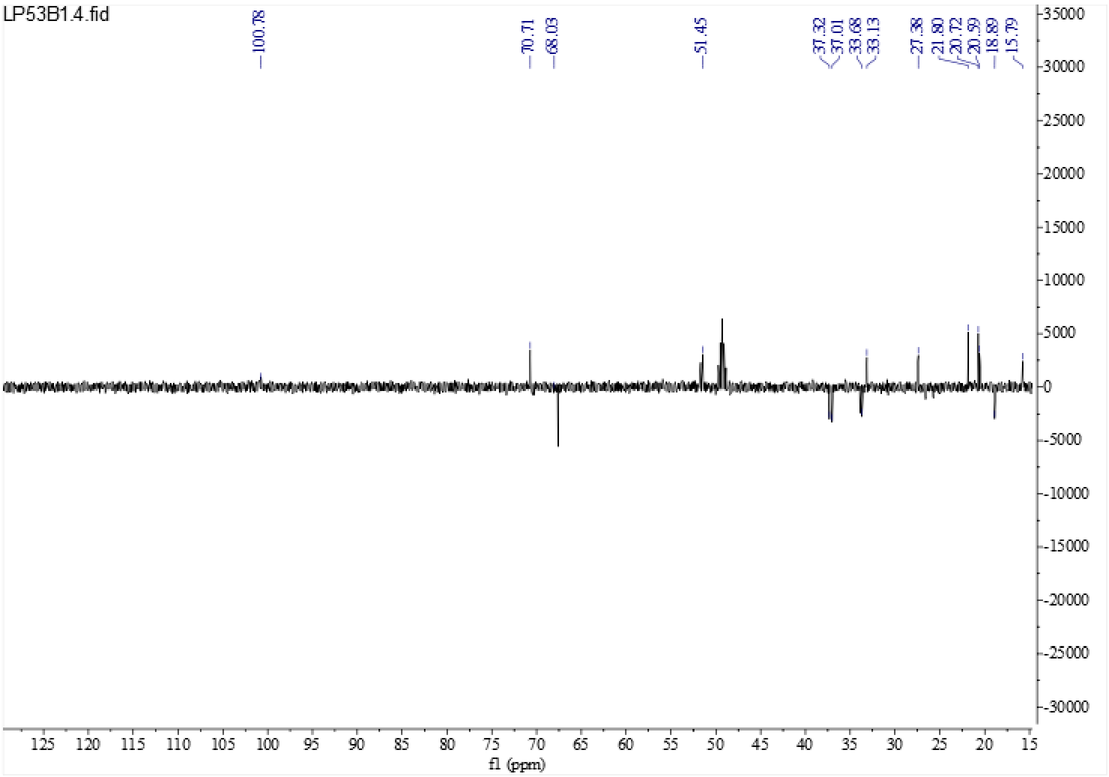

Figure S23 The DEPT Spectrum of Compound **3** in CD_3OD.

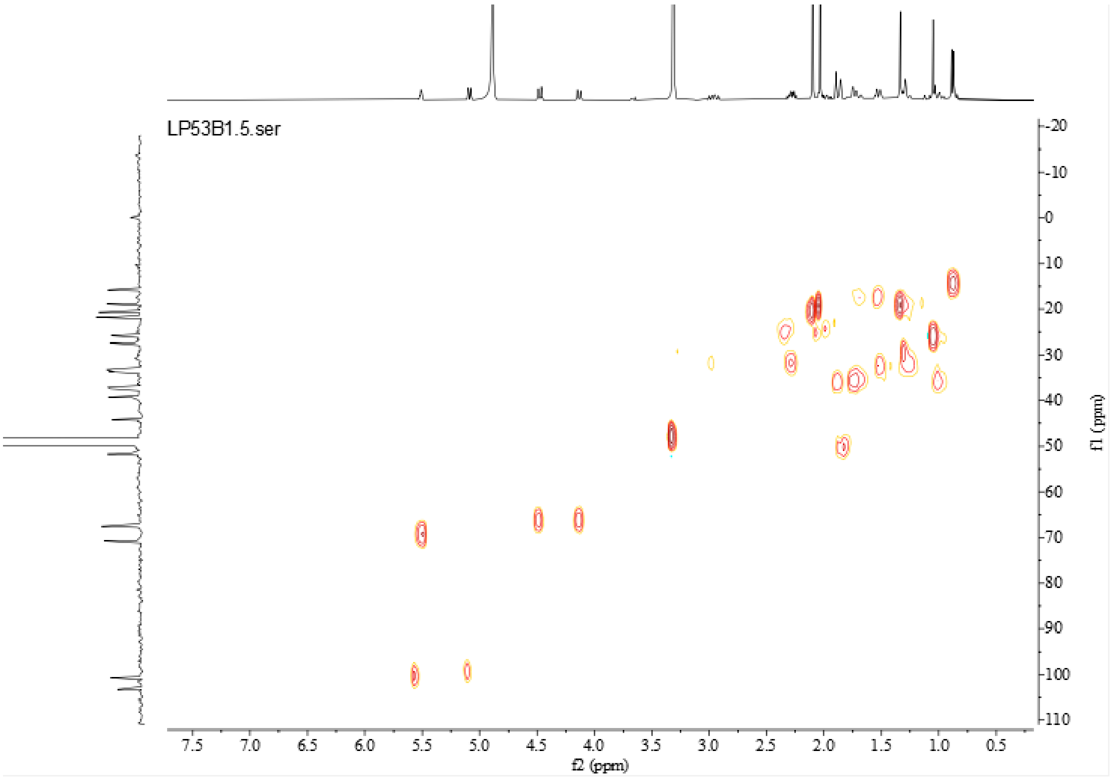

Figure S24 The HSQC Spectrum of Compound **3** in CD_3OD.

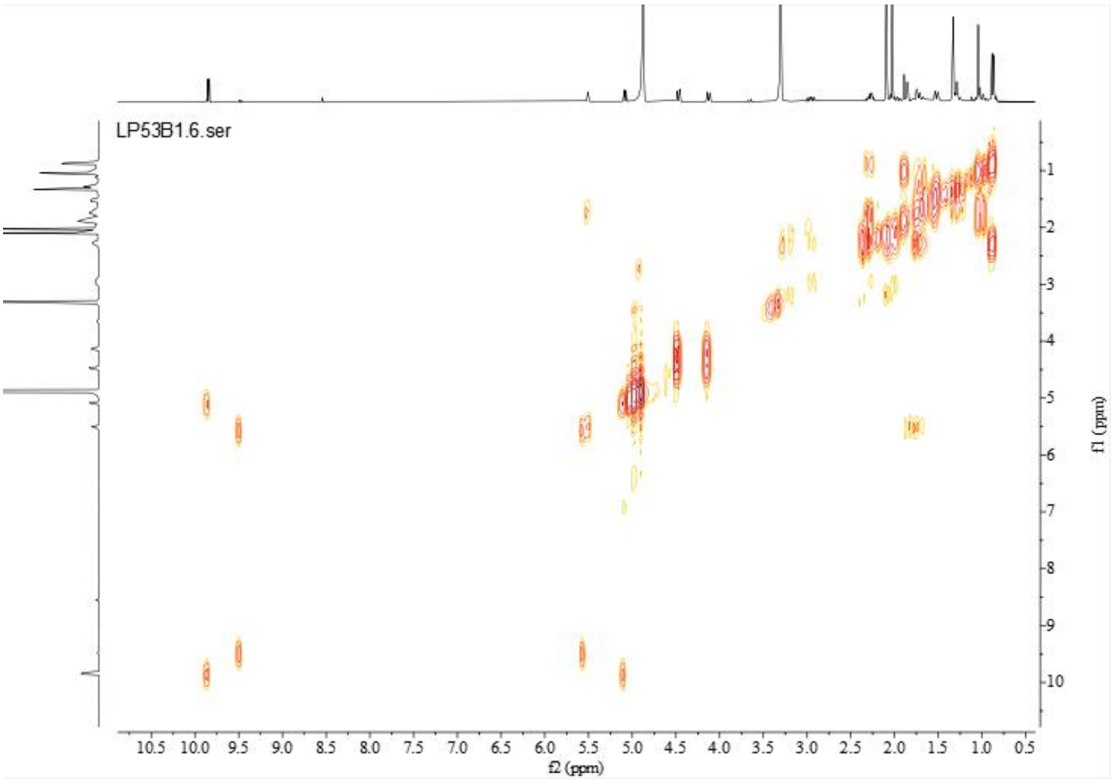

Figure S25 The ^{1}H-^{1}H COSY Spectrum of Compound **3** in CD_3OD.

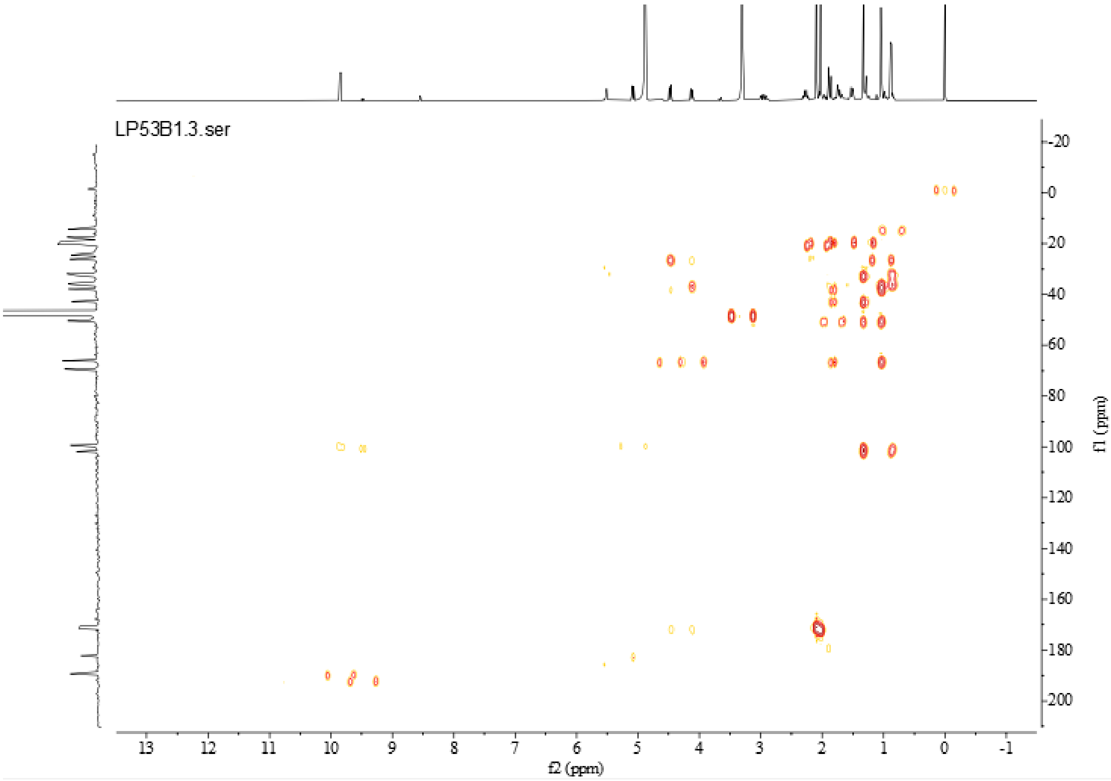

Figure S26 The HMBC Spectrum of Compound **3** in CD_3OD.

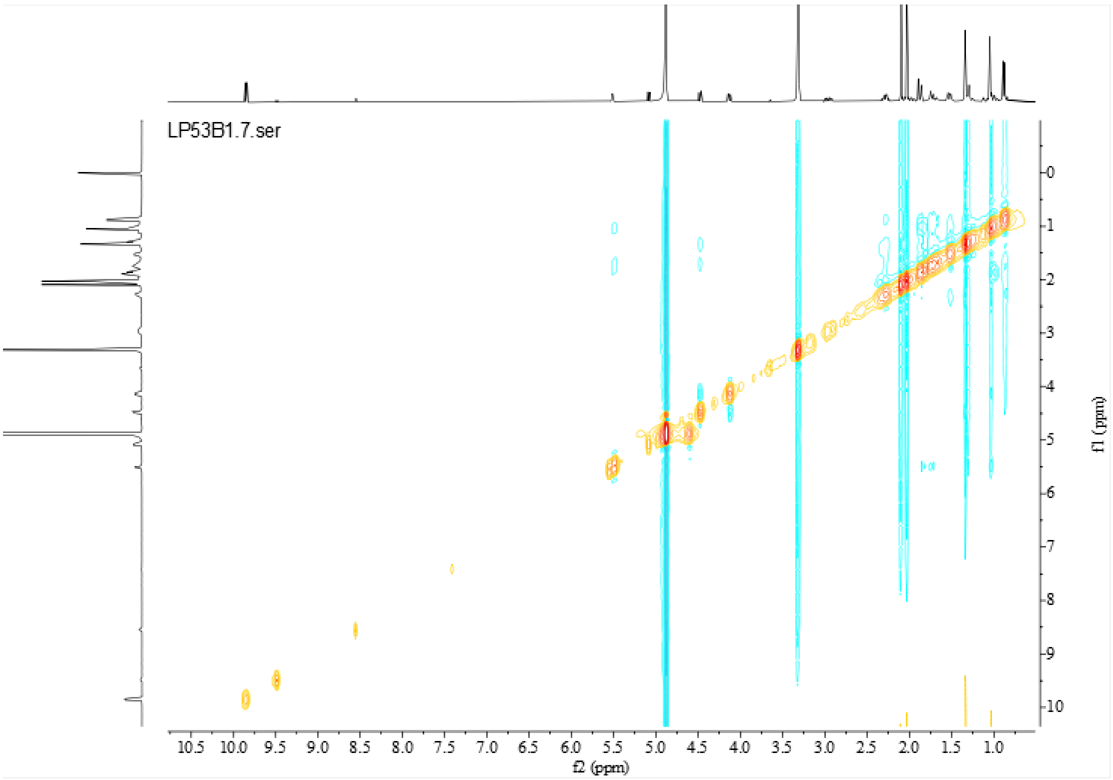

Figure S27 The NOESY Spectrum of Compound **3** in CD_3OD.

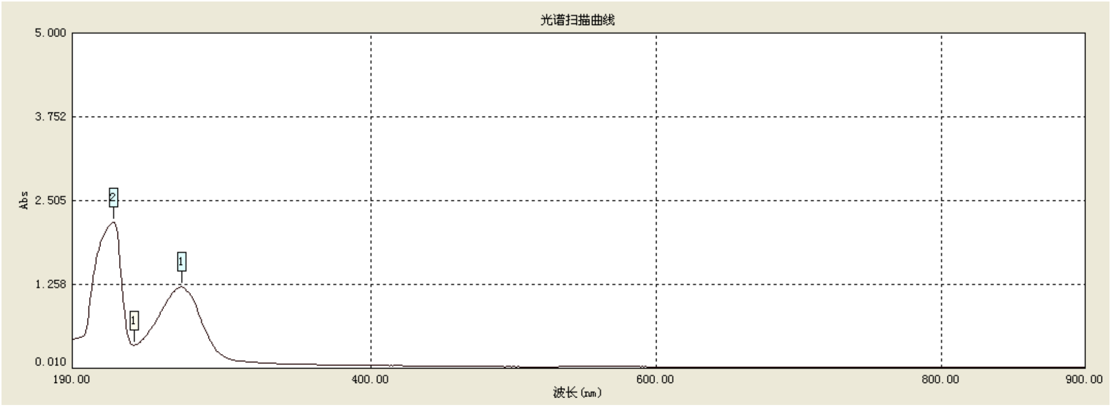

Figure S28 The UV Spectrum of Compound **4**.

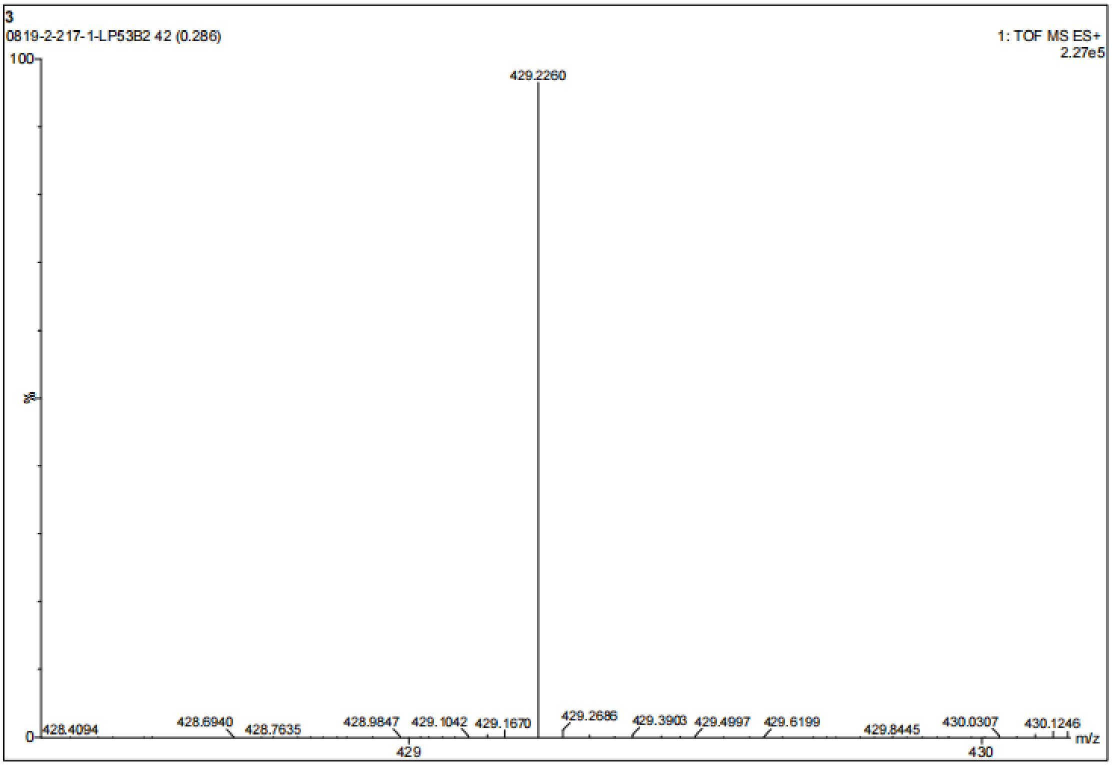

Figure S29 The (+)-HRMS(ESI) Spectroscopic Data of Compound **4**.

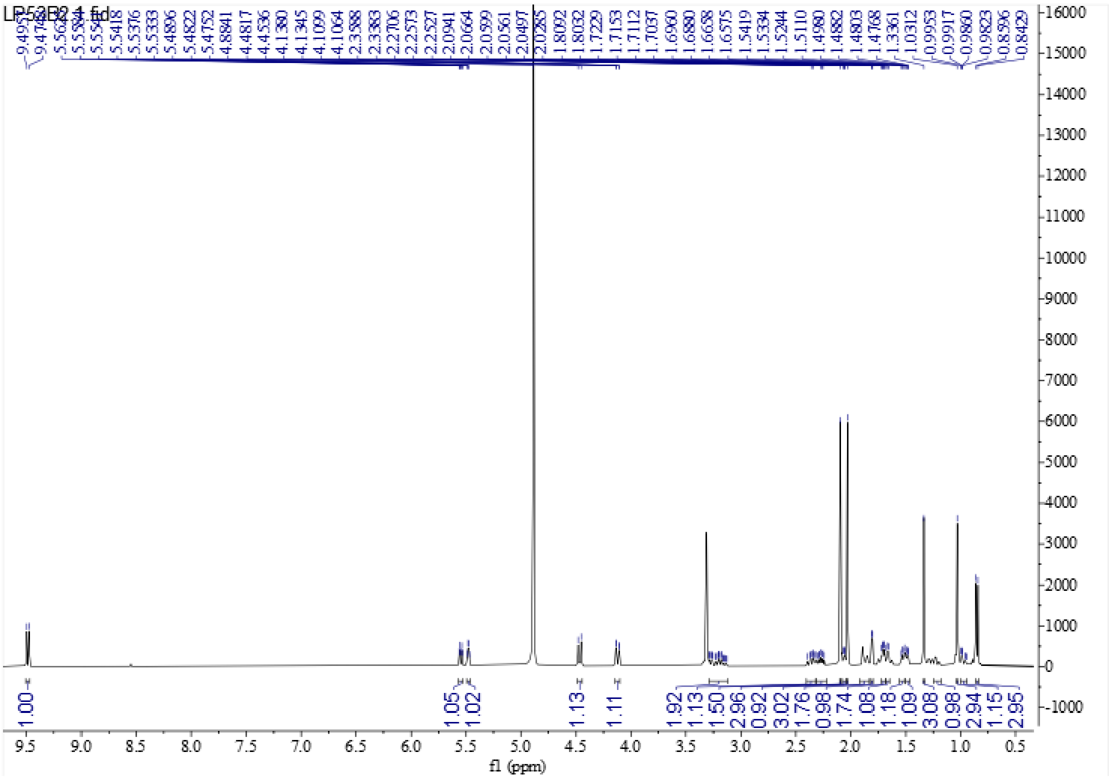

Figure S30 The ^{1}H NMR Spectrum of Compound **4** in CD_3OD.

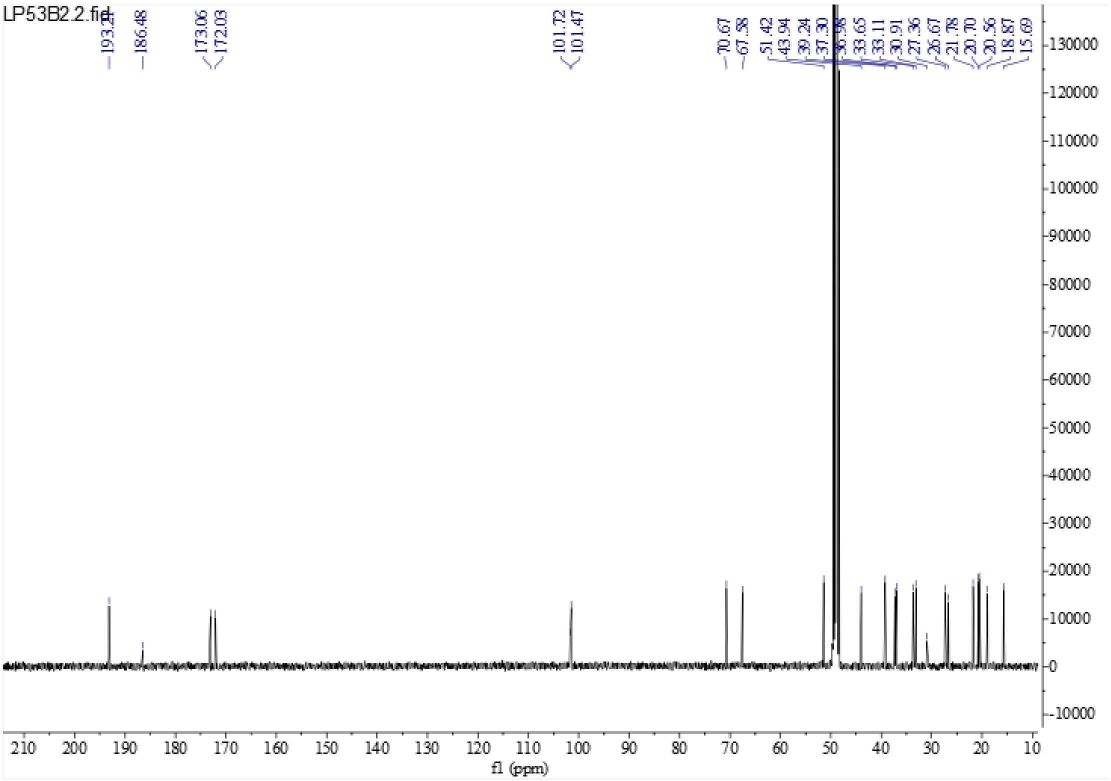

Figure S31 The ^{13}C NMR Spectrum of Compound **4** in CD_3OD.

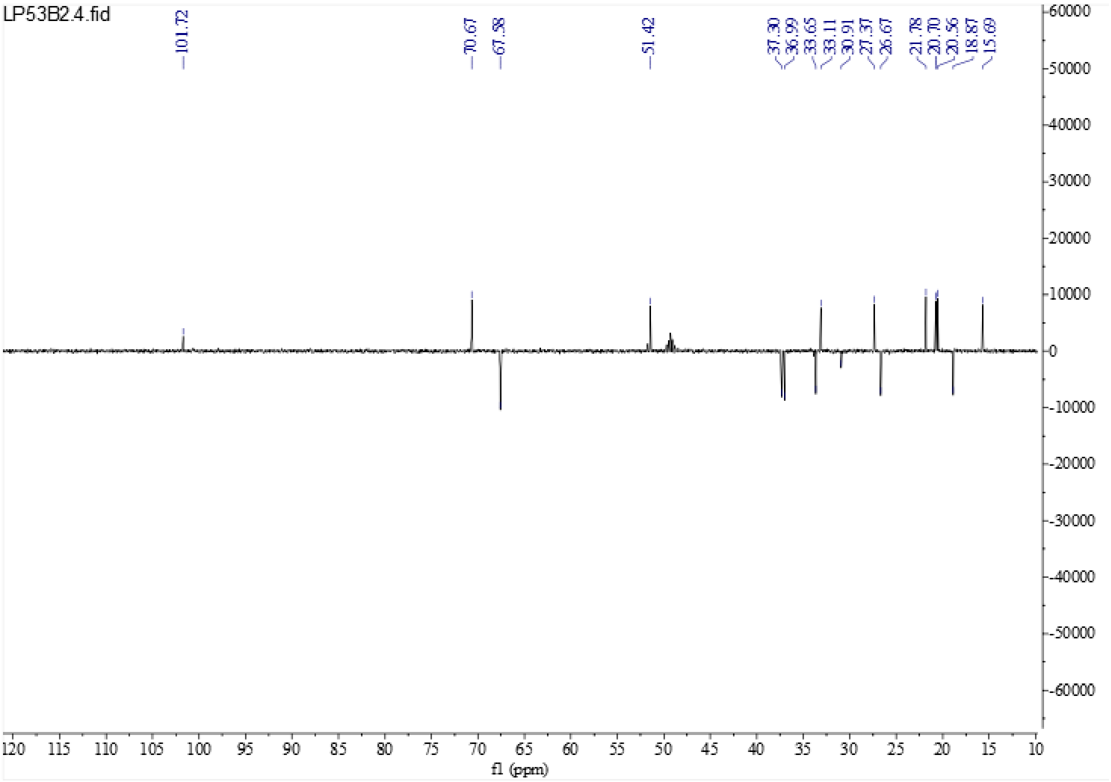

Figure S32 The DEPT Spectrum of Compound **4** in CD_3OD.

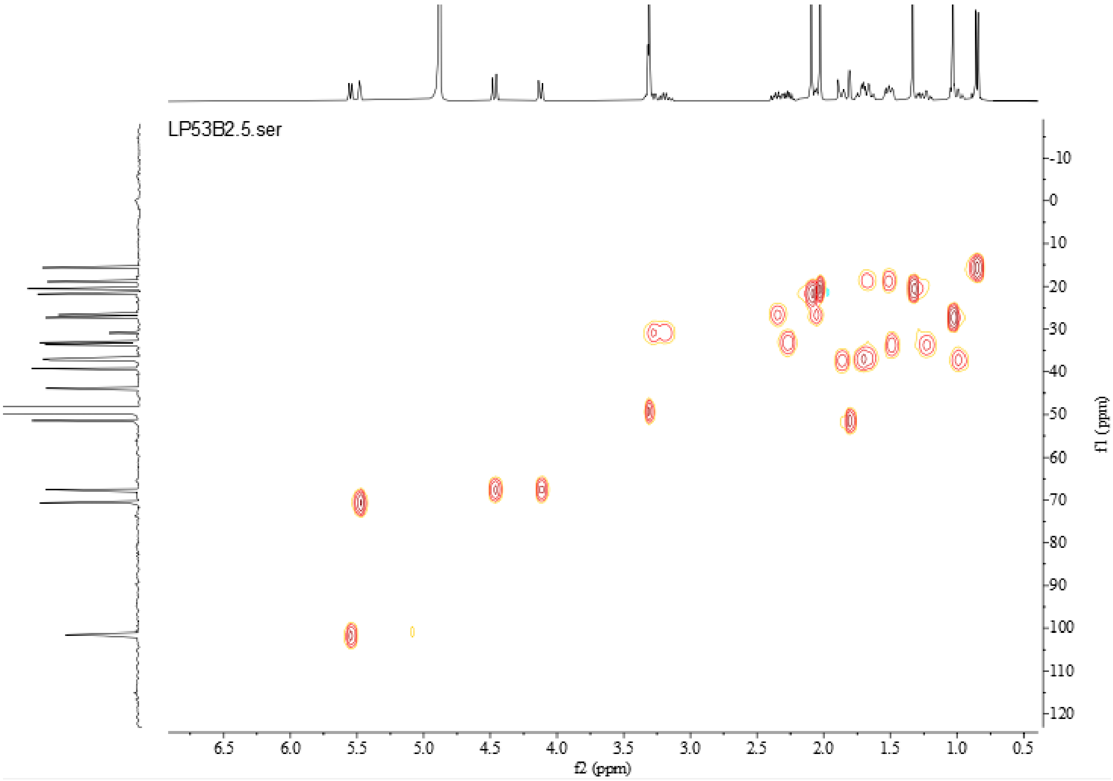

Figure S33 The HSQC Spectrum of Compound **4** in CD_3OD.

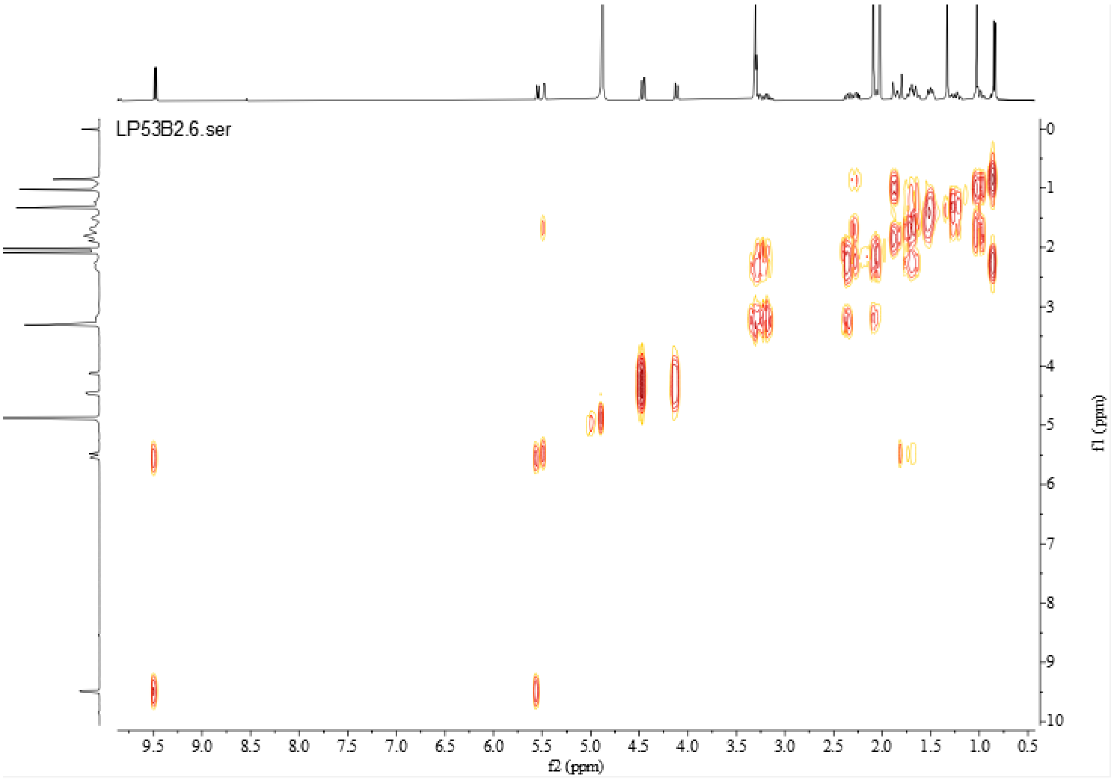

Figure S34 The ^1H-^1H COSY Spectrum of Compound **4** in CD_3OD.

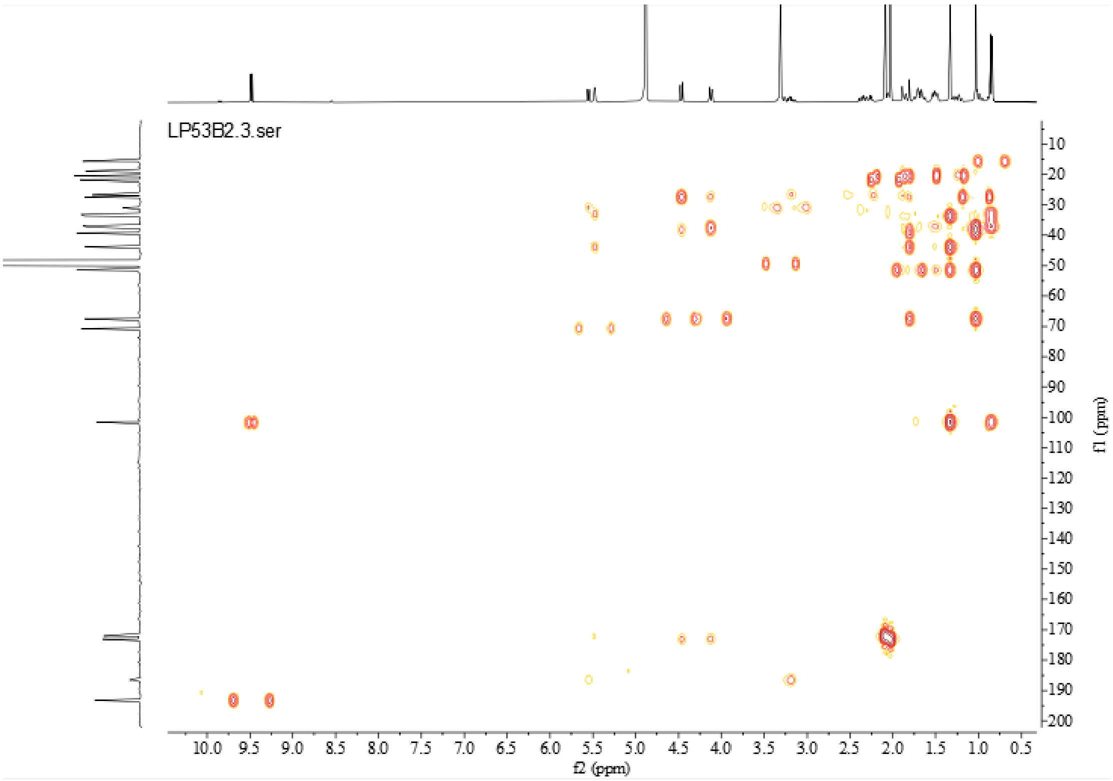

Figure S35 The HMBC Spectrum of Compound **4** in CD_3OD.

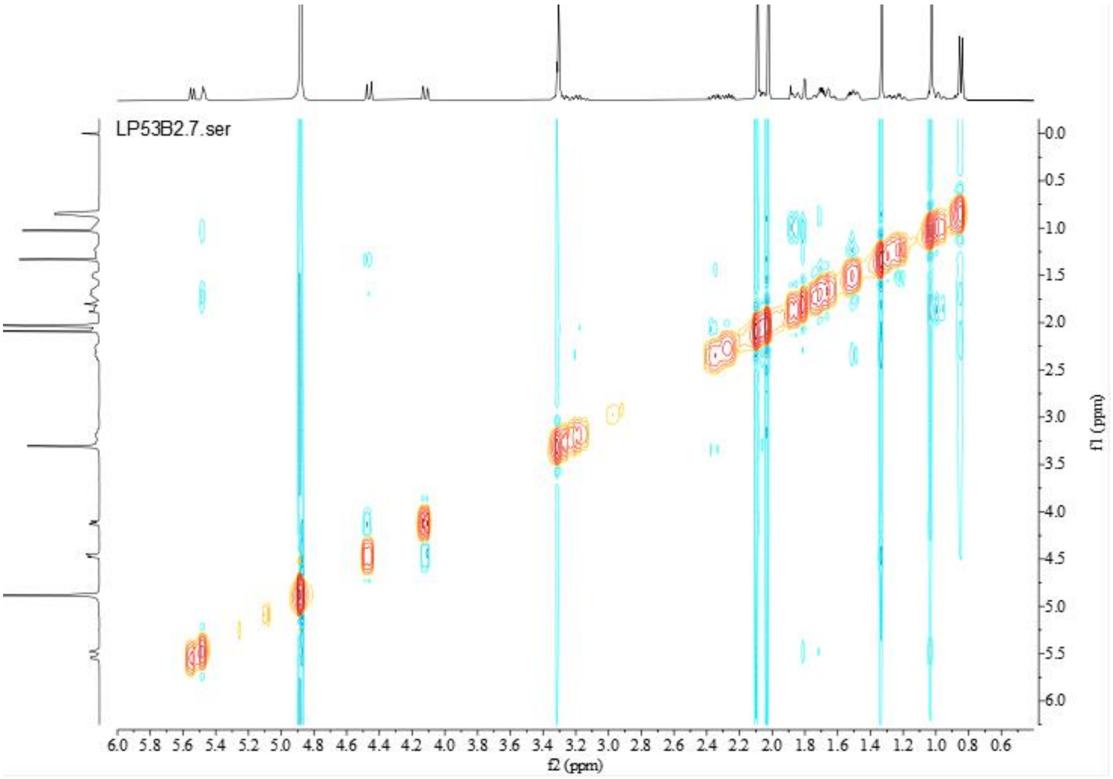

Figure S36 The NOESY Spectrum of Compound **4** in CD_3OD.
